# Chitosan-encapsulated manganese ferrite particles bearing sulfonic acid group catalyzed efficient synthesis of spiro indenoquinoxalines†

**DOI:** 10.1039/d0ra04925e

**Published:** 2020-09-09

**Authors:** Sepideh Lahouti, Hossein Naeimi

**Affiliations:** Department of Organic Chemistry, Faculty of Chemistry, University of Kashan Kashan Iran naeimi@kashanu.ac.ir +98 591 2397 +98 591 2388

## Abstract

A simple, highly versatile and efficient synthesis of 5-phenyl-spiro[diindenopyridine-indenoquinoxaline]-diones is achieved through a four-component one pot reaction of ninhydrin, 1,2-diaminobenzene, 1,3-indandione and aniline. This reaction was catalyzed by MnFe_2_O_4_@CS-Bu-SO_3_H MNPs as a very efficient, recyclable heterogeneous catalyst in acetonitrile under reflux conditions. The catalyst can be recovered from the subsequent reaction mixture and reused for at least five cycles without any appreciable loss in its efficiency. The core–shell structure and composition of the produced magnetic nanocatalyst were analyzed using FT-IR, XRD, VSM, SEM, EDX and TGA techniques.

## Abstract 2

A simply, highly versatile and efficient synthesis of 5-phenyl-spiro [diindeno pyridineindenoquinoxaline]-diones is achieved through five component one pot reaction of ninhydrin, 1,2-diaminobenzene, 1,3-indandione and anilines.

## Graphical abstract

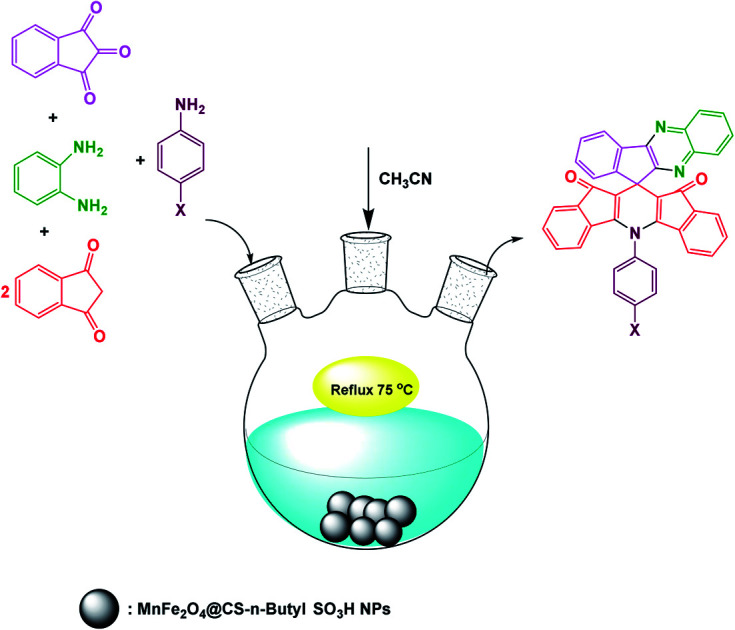

## 1. Introduction

Spirocycles, structures that have two or more rings joined at a single carbon, remain a challenging design for synthetic chemists. Some of the most important spirocycles are spirooxindole and spiroindoline alkaloids isolated from natural sources.^1–3^ Spiropyrans and spiroquinoxalines, in particular, have possible applications as photochromic materials in data recording, storage, and transfer and in other industrial fields.^4^ The indole nucleus is perhaps the most well-known heterocycle, a common and important feature of a variety of natural products and medicinal agents.^5^ The spirooxindole ring system is a broadly dispersed structural framework present in a number of natural and pharmaceutical products. Indenoquinoxaline derivatives are an important class of nitrogen-containing heterocycles which are useful intermediates in organic synthesis.^6,7^

Heterogeneous catalysis has been studied for many years and has become vital for efficient and eco-friendly organic transformations over the past few decades.^8–10^ Magnetite is an ideal oxide support and easy to make, with a very effective surface for adsorptions or immobilization of metals and ligands, which can be separated by magnetic separation.^11^ Magnetite nanoparticles are not very resistant under ambient conditions and are easily oxidized to magnetite or dissolved in an acidic medium.^12^

Chitosan (CS) has various favorable properties, such as, biocompatibility, low toxicity, good film making, high mechanical strength and high hydrophilicity, and has therefore been an important material for the preparation of magnetic carriers.

Chitosan is a natural attractive due to the presence of amine and hydroxyl groups.^13–16^ These groups serve as adsorption sites for many adsorbates. Therefore, it is an appropriate polymer for use to modify MnFe_2_O_4_ NPs. Many investigations have been performed on chitosan-modified magnetic NPs for biomedical applications.^17^

Multicomponent reactions (MCRs) enjoy an outstanding reputation in organic and medicinal chemistry for their high degree of atomic economy and application in the diversity oriented convergent synthesis of complex organic molecules from simple and readily available layers in a single vessel; they fulfil some of the objectives of perfect synthesis because complex products are formed in a single step and diversity can be obtained simply by varying the reaction components.^18–24^

**Scheme 1:**
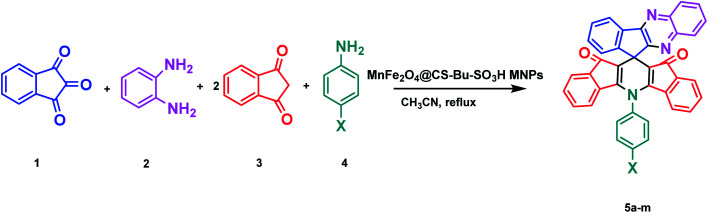
Synthesis of 5-phenyl-spiro[diindenopyridine-indenoquinoxaline]-dione using MnFe_2_O_4_@CS-Bu-SO_3_H MNPs.

In continuation of our previously reported work on catalytic reactions,^25,26^ herein we report the preparation of MnFe_2_O_4_@CS-*n*-Bu-SO_3_H MNPs and their use as an efficient nanocatalyst for the synthesis of 5-phenyl-spiro[diindenopyridine-indenoquinoxaline]-dione derivatives through the condensation of ninhydrin, 1,2-diamino-benzene, 1,3-indandione and various anilines under thermal conditions (Scheme 1).

## 2. Results and discussion

### 2.1. Preparation and characterization of MnFe_2_O_4_@CS-Bu-SO_3_H MNPs

The manganese ferrite nanoparticles were prepared by co-precipitation of Fe(iii) and Mn(ii) at a molar ratio of 2 : 1 which was dropped slowly into NaOH solution at a temperature of 97 °C. Then, the synthesized magnetic nanoparticles were modified with chitosan as a natural polymer. To prepare the modified MnFe_2_O_4_–chitosan nanoparticles, MnFe_2_O_4_ nanoparticles were dispersed in distilled water under ultrasound irradiation and chitosan in acetic acid solution was slowly added under vigorous stirring for 1 h. The magnetically heterogeneous organocatalyst MnFe_2_O_4_@CS-Bu-SO_3_H MNPs were characterized by X-ray diffraction (XRD), scanning electron microscopy (SEM), energy-dispersive X-ray spectroscopy (EDX), vibrating sample magnetometry (VSM), thermal gravimetric analysis (TGA) and Fourier transform infrared (FT-IR) spectroscopy.

#### 2.1.1 FT-IR analysis

**Fig. 1:**
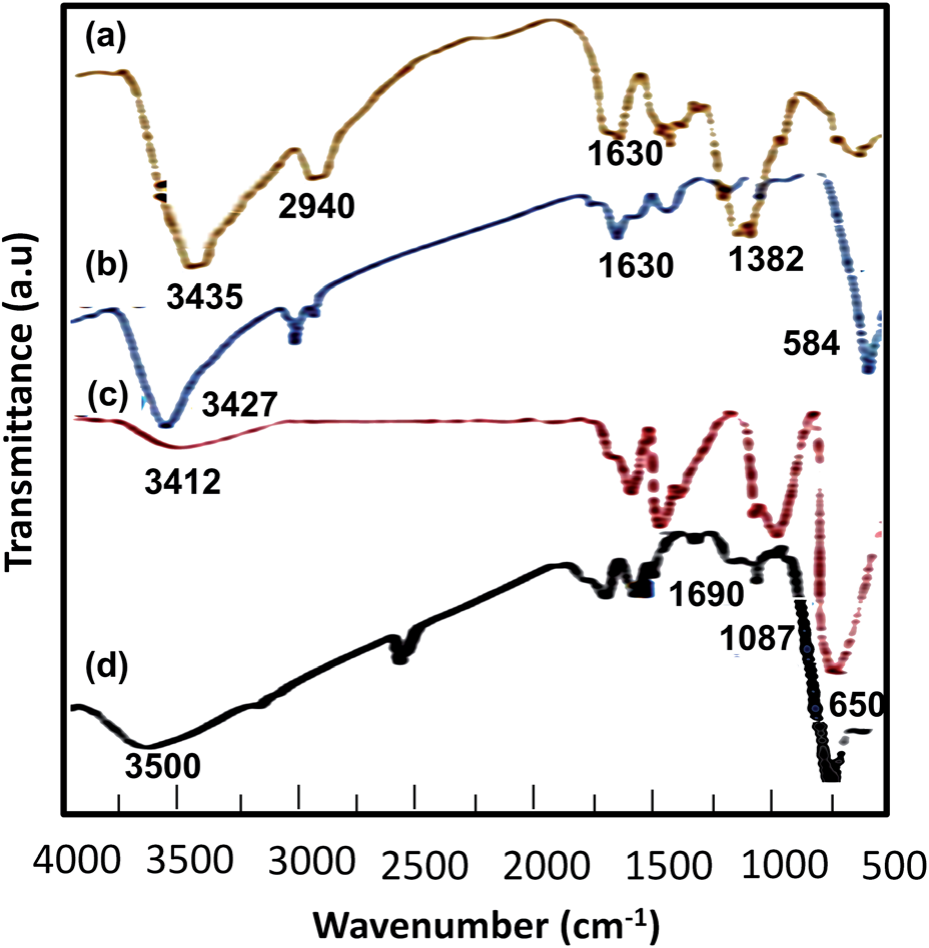
IR spectra of (a) chitosan (CS), (b) MnFe_2_O_4_, (c) MnFe_2_O_4_@CS NPs and (d) MnFe_2_O_4_@CS-Bu-SO_3_H MNPs.

The FT-IR spectra (Fig. 1) of (a) chitosan, (b) MnFe_2_O_4_, (c) MnFe_2_O_4_@CS NPs and (d) MnFe_2_O_4_@CS-Bu-SO_3_H MNPs are instructive. The O–H stretching vibration was detected at 3428 and 3433 cm^−1^ in the peaks of Fig. 1a–d. The FT-IR spectrum of chitosan was characterized by the following absorption bands: the (NH) of the polymer backbone at 3435 and 1630 cm^−1^, the (C–O) of the primary alcoholic group at 1382 cm^−1^, and (C–H) at 2940 cm^−1^ (Fig. 1a). The FT-IR spectrum of MnFe_2_O_4_ showed characteristic peaks at 3427 cm^−1^ and 1630 cm^−1^ which were assigned to the vibration of hydroxyl groups; in addition, an obvious peak at 584 cm^−1^ was attributed to the Fe–O bond vibration (Fig. 1b). Compared with curve b, curve c shows the most significant band shift of Fe–O stretching (from 584 to 657 cm^−1^), indicating that iron ions bind to the NH_2_ group of chitosan. Electrostatic interaction between the negatively charged MnFe_2_O_4_ nanoparticle surface and the positively charged protonated chitosan also contributes to the IR change. The peak at 3412 cm^−1^ was probably the amino group of chitosan, which is overlapped by the O–H stretching vibration of MnFe_2_O_4_ nanoparticles (Fig. 1c). The FT-IR spectrum of MnFe_2_O_4_@CS-Bu-SO_3_H MNPs shows the stretching and out-of-plane bending of acidic O–H groups as two broad bands at 2800–3500 cm^−1^. Also, the S=O stretching bands of the –SO_3_H group appeared at 1000–1200 cm^−1^ (Fig. 1d).

#### 2.1.2 X-ray diffraction (XRD) analysis

**Fig. 2:**
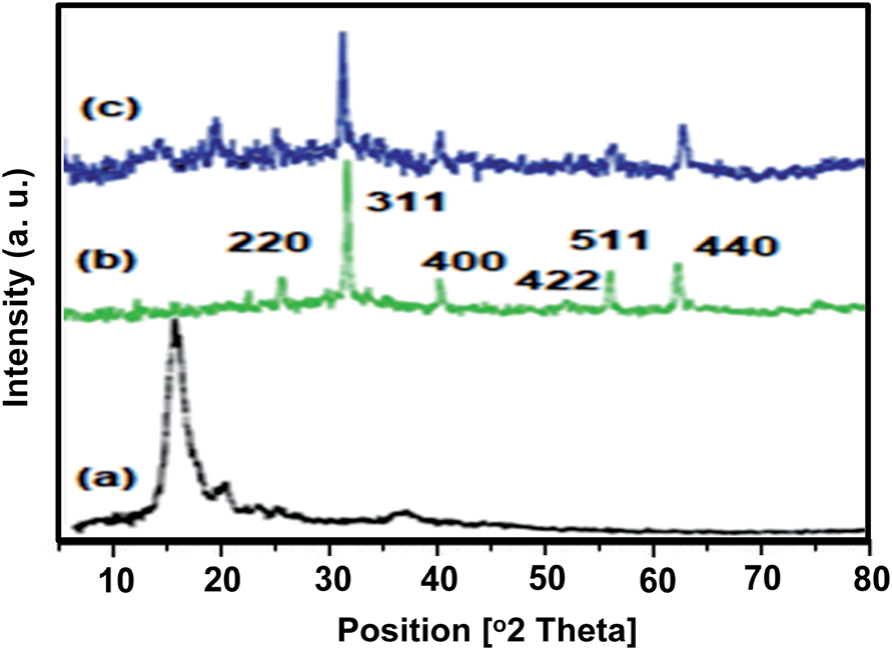
XRD patterns of (a) chitosan (CS), (b) MnFe_2_O_4_, and (c) MnFe_2_O_4_@CS-Bu-SO_3_H MNPs.

The crystal structures of chitosan and MnFe_2_O_4_@CS-*n*-Bu-SO_3_H MNPs are shown in Fig. 2. The sharp peaks in the XRD patterns confirm the good crystallinity of the prepared samples. There are six diffraction peaks at 2*θ* values of about 29.94°, 35.24°, 42.75°, 52.94°, 56.42° and 61.86°, corresponding to the (220), (311), (400), (422), (511), and (440) planes in the MnFe_2_O_4_@CS-*n*-Bu-SO_3_H MNPs, which is the standard pattern for crystalline magnetite with cubic structure (JCPDS card no. 01-1111). The small and weak broad bands in the range of 12–20° show the existence of amorphous chitosan. The average crystal sizes of pure MnFe_2_O_4_ and chitosan-coated MnFe_2_O_4_ nanoparticles are 19 and 25, respectively.

#### 2.1.3 Magnetic properties

**Fig. 3:**
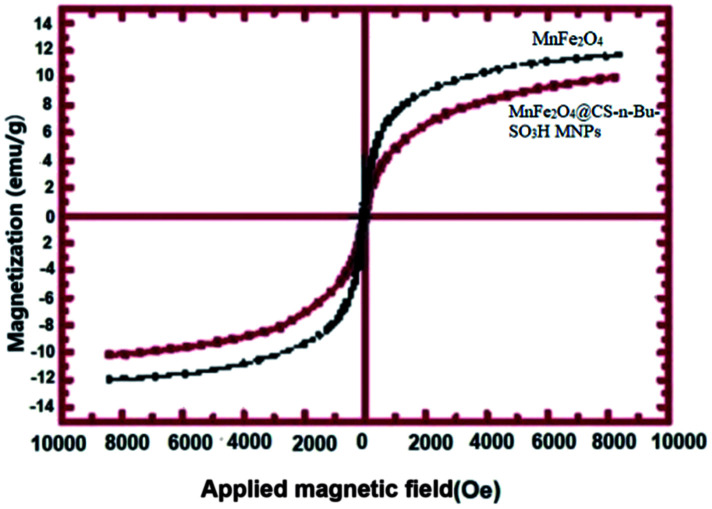
Magnetization *versus* applied field for MnFe_2_O_4_ and MnFe_2_O_4_@CS-Bu-SO_3_H MNPs.

The magnetization curves for MnFe_2_O_4_ nanoparticles and MnFe_2_O_4_@CS-Bu-SO_3_H MNPs nanoparticles are shown in Fig. 3. Room temperature specific magnetization *versus* applied magnetic field curve measurements of the MnFe_2_O_4_@CS-Bu-SO_3_H MNPs indicate a saturation magnetization value of 10.27 emu g^−1^, lower than that of the pristine MnFe_2_O_4_ nanoparticles (12.69 emu g^−1^) due to the coated shell.

#### 2.1.4 Thermal gravimetric analysis (TGA)

**Fig. 4:**
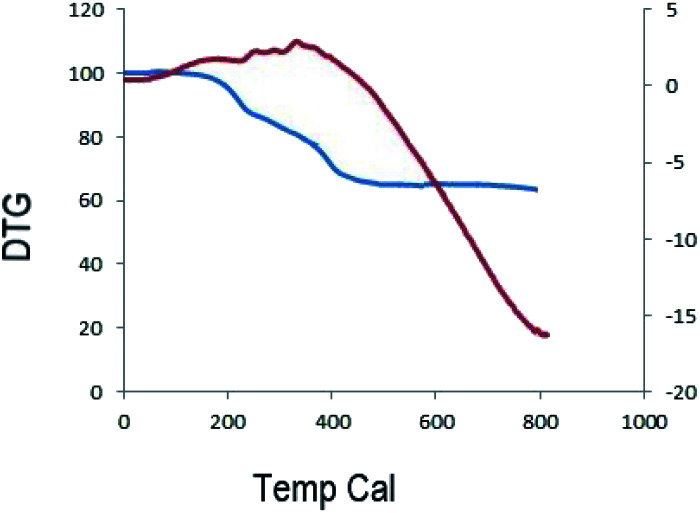
The thermal gravimetric analysis (TGA) of MnFe_2_O_4_@CS-Bu*-*SO_3_H NPs.

In order to obtain information on the thermal stability, TGA experiments were carried out by heating MnFe_2_O_4_@CS-Bu-SO_3_H MNPs up to 800 °C (Fig. 4). The weight loss of MnFe_2_O_4_@CS-Bu-SO_3_H MNPs nanoparticles is about 40% in the range 250–420 °C, corresponding to the thermal decomposition of the chitosan chains over MnFe_2_O_4_ nanoparticles.

#### 2.1.5 Scanning electron microscopy (SEM)

**Fig. 5:**
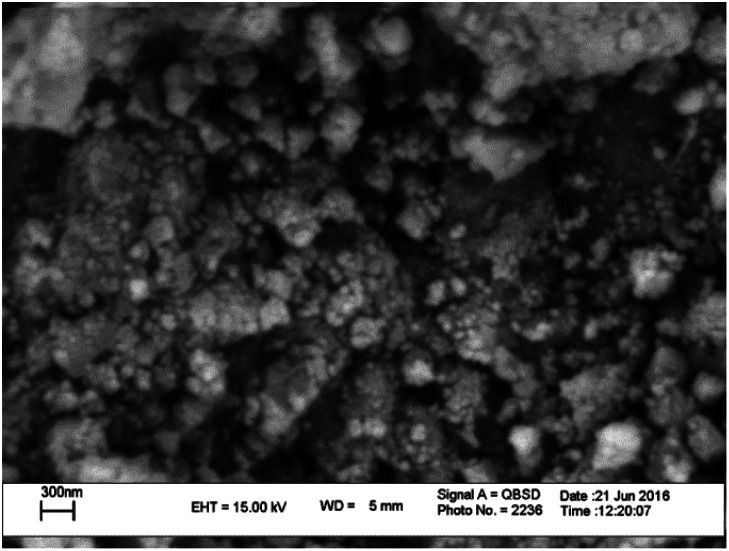
SEM image of MnFe_2_O_4_@CS-Bu-SO_3_H MNPs.

Fig. 5 exhibits the morphology and particle size of MnFe_2_O_4_@CS-Bu-SO_3_H MNPs. It indicates that the chitosan polymeric matrix uniformly covers the surface of MnFe_2_O_4_ (Fig. 5). The SEM image shows that the structural size of MnFe_2_O_4_@CS-Bu-SO_3_H MNPs is bigger. The SEM of MnFe_2_O_4_@CS-Bu-SO_3_H MNPs clearly reveals the structure of the CS-coated magnetite nanoparticles.

#### 2.1.6 Energy dispersive X-ray (EDX) spectroscopy

**Fig. 6:**
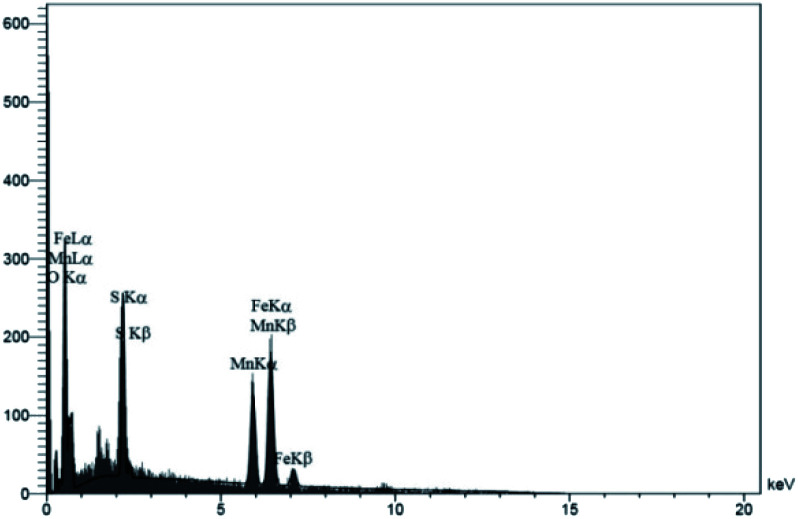
The energy dispersive X-ray (EDX) spectrum of MnFe_2_O_4_@CS-Bu-SO_3_H MNPs.

The elemental compositions are calculated from the energy dispersive X-ray (EDX) spectrum. The elemental composition of MnFe_2_O_4_@CS-Bu-SO_3_H MNPs is 3.01%, 11.56%, 6.83%, 29.73%, 30.74% and 18.12% for N, C, S, O, Fe and Mn, respectively. This implies that the chitosan polymer was coated on the surface of the MnFe_2_O_4_ NPs (Fig. 6).

**Fig. 7:**
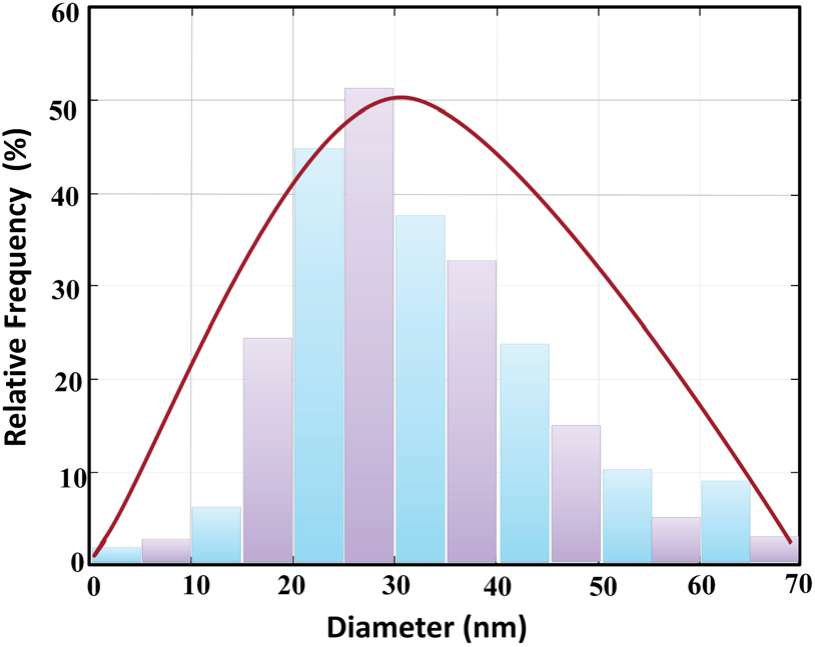
Particle size distribution histogram of the catalyst.

Moreover, in accordance with the SEM image of the MnFe_2_O_4_@CS-Bu-SO_3_H nanoparticles, the particle size distribution histogram is shown in Fig. 7. As can be observed, the dispersed nanoparticles formed with a uniform size and the mean value and standard deviation could be estimated as 27 ± 3 nm from this size distribution histogram.

### 2.2. Investigation of catalyst activity in organic reactions

In the present work, we investigated the model reaction of ninhydrin, 1,2-diamino benzene, 1,3-indandione and anilines in the presence of MnFe_2_O_4_@CS-Bu-SO_3_H MNPs under heat condition. In this research, the effects of different solvents and various amounts of catalyst on the model reaction were examined. First, several organic solvents, such as H_2_O, CH_3_CN, EtOH and CHCl_3_, were examined (Table 1). According to the data given in Table 1, entry 3, CH_3_CN was the most efficient solvent for this reaction.

**Table 1:** The reaction in the presence of different solvents and MnFe_2_O_4_@CS-Bu-SO_3_H MNPs^a^

Entry	Solvent	Time (h)	Yield^b^ (%)
1	H_2_O	18	38
2	EtOH	15	43
3	CH_3_CN	3	97
4	CHCl_3_	24	31

a Reaction conditions: ninhydrin 1.0 mmol, 1,2-diamino benzene 1.0 mmol, 1,3-indandione 2.0 mmol and anilines 1.0 mmol under heat.

b Isolated yields.

Furthermore, various catalysts and different amounts of catalyst were used in the sample reaction (Table 2). No reaction was observed in the absence of catalyst without heat (Table 2, entry 1). When the reaction was performed at room temperature in the presence of MnFe_2_O_4_@CS-Bu-SO_3_H MNPs, the product was obtained in lower yield and with longer reaction time (Table 2, entry 6), while heat and MnFe_2_O_4_@CS-*n*-butyl SO_3_H NPs gave the best yield in the shortest time. An excellent yield of 97% was obtained with 20 mol% catalyst (Table 2, entry 8) and was not improved by increasing to 30 mol% (Table 2, entry 9).

**Table 2:** Optimization of the various amounts and types of catalysts^a^

Entry	Condition	Catalyst (mol%)	Time (h)	Yield^b^ (%)
1	R.T.	—	48	—
2	Heat	Et_3_N	24	10
3	Heat	*p*-TSA	16	70
4	Heat	Nano Fe_3_O_4_	20	43
5	Heat	Nano MnFe_2_O_4_	18	48
6	R.T.	MnFe_2_O_4_@CS-Bu-SO_3_H NPs (10)	48	10
7	Heat	MnFe_2_O_4_@CS-Bu-SO_3_H NPs (10)	3	76
8	Heat	MnFe_2_O_4_@CS-Bu-SO_3_H NPs (20)	3	97
9	Heat	MnFe_2_O_4_@CS-Bu-SO_3_H NPs (30)	3	97

a Reaction conditions: ninhydrin 1.0 mmol, 1,2-diamino benzene 1.0 mmol, 1,3-indandione 2.0 mmol and anilines 1.0 mmol.

b Isolated yields.

We also applied MnFe_2_O_4_@CS-Bu-SO_3_H MNPs catalyst for the synthesis of 5-phenyl-spiro[diindenopyridine-indenoquinoxaline]-dione derivatives from various aromatic amines under similar conditions, as seen in Table 3. The results indicate that excellent yields were achieved in the reaction in the presence of MnFe_2_O_4_@CS-Bu-SO_3_H MNPs (20 mol%) under reflux conditions. The influence of electron-withdrawing and electron-donating substituents on the aromatic ring of amines upon the reaction yields was investigated. Electron-releasing substituents on aromatic amines gave good to excellent yields of 5-phenyl-spiro[diindenopyridine-indenoquinoxaline]-dione derivatives under the above-mentioned conditions. The highest yields of 5-phenyl-spiro[diindenopyridine-indenoquinoxaline]-dione derivatives were obtained using the electron-releasing para-methyl group as a substituent for aromatic amines.

**Table 3:** Synthesis of various 5-phenyl-spiro[diindenopyridine-indenoquinoxaline]-dione derivatives using MnFe_2_O_4_@CS-Bu-SO_3_H MNPs^a^

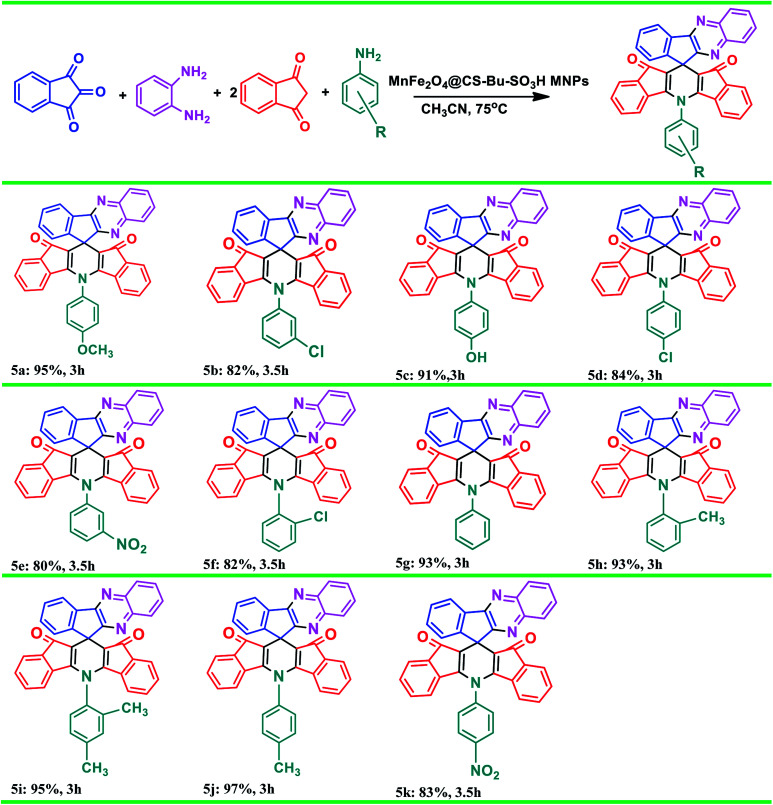

a Isolated yield.

Furthermore, comparison of the reactions in the presence of *p*-TSA catalyst, as previously reported,^6^ with the present work was carried out on production of 5a, 5j and 5k. The results are listed in Table 4. As shown, the product yields using the present method were higher than those from the previously reported method and the reaction times were shorter than those obtained in the reported work (Table 4, entries 2, 4, 6 *vs.* 1, 3, 5).

**Table 4:** Comparison between previously reported work and the present work

En.	Catalyst	Cond.	Prod.	Time (h)	Yield (%)	Ref.
1	*p*-TSA (30 mol%)	H_3_CCN, 82 °C	5a	16	71	6
2	MnFe_2_O_4_@CS-Bu-SO_3_H	H_3_CCN, 75 °C	5a	3	95	This work
3	*p*-TSA (30 mol%)	H_3_CCN, 82 °C	5j	16	95	6
4	MnFe_2_O_4_@CS-Bu-SO_3_H	H_3_CCN, 75 °C	5j	3	97	This work
5	*p*-TSA (30 mol%)	H_3_CCN, 82 °C	5k	16	62	6
6	MnFe_2_O_4_@CS-Bu-SO_3_H	H_3_CCN, 75 °C	5k	3.5	83	This work

**Scheme 2:**
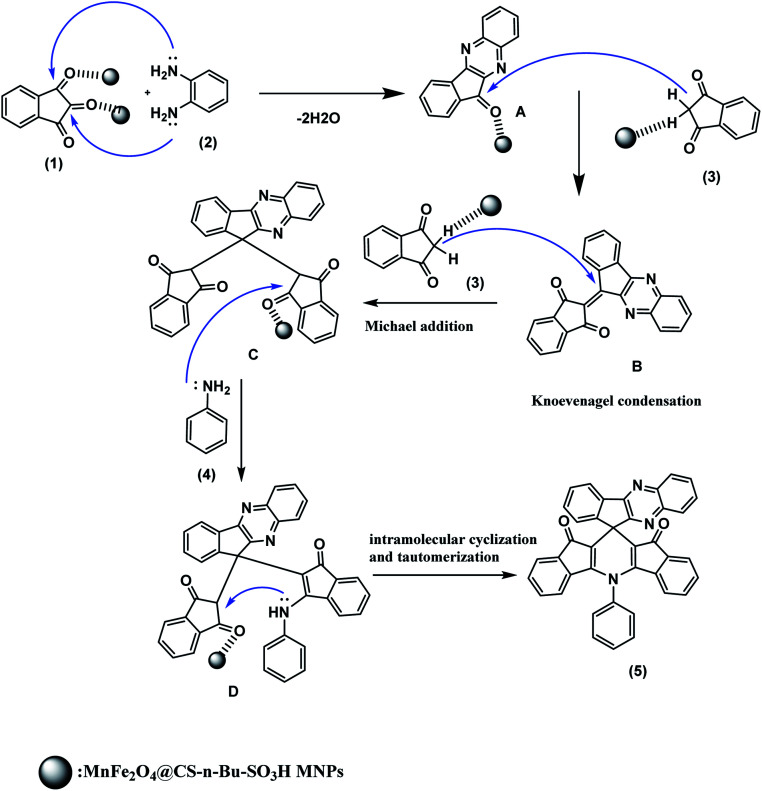
Plausible mechanism for the synthesis of spiro indenoquinoxalines.

The proposed reaction mechanism for the synthesis of spiro indenoquinoxalines catalyzed by MnFe_2_O_4_@CS-*n*-Bu-SO_3_H MNPs is depicted in Scheme 2. As shown in Scheme 2, compound 5 could be synthesized *via* sequential iminization–aromatization, condensation, addition, enamination and cyclization. Initially, the MnFe_2_O_4_@CS-*n*-Bu-SO_3_H MNPs catalyst protonates the carbonyl group of ninhydrin (1); this step is regarded as a fast iminization–aromatization reaction in which the ninhydrin (1) first reacts with 1,2-diamino benzene (2) to afford 11*H* indeno[2,1-*b*]quinoxalin-11-one (A) in the presence of MnFe_2_O_4_@CS-Bu-SO_3_H MNPs in acetonitrile. Then, the catalyst protonates the carbonyl group of compound A and compound A is condensed with 1,3-indenedione (3) to afford compound B. This step is regarded as a fast Knoevenagel condensation reaction. Then, compound B is attacked by another 1,3-indene dione (3) in a Michael-type addition to produce intermediate C. Finally, compound C reacted with aniline (4) to produce compound D, followed by intramolecular cyclization and tautomerization to afford the product (5).

**Fig. 8:**
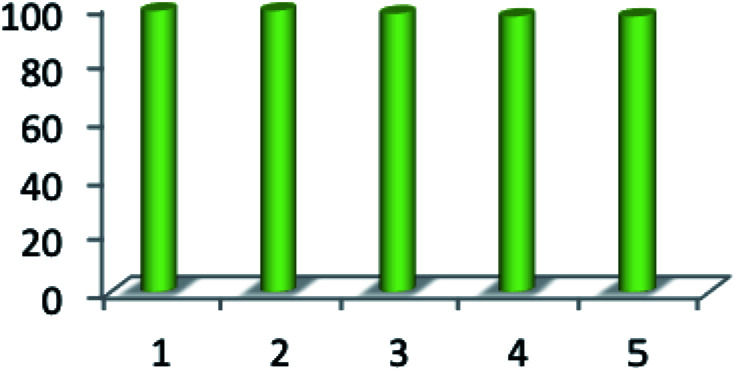
Reusability of catalyst for synthesis of spiro indenoquinoxaline.

Reusability is one of the significant properties of this catalyst. The reusability of MnFe_2_O_4_@CS-Bu-SO_3_H MNPs was studied for the reaction of ninhydrin, 1,2-diamino benzene, 1,3-indandione and anilines. After completion of the reaction, the nanocatalyst was easily separated using an external magnet. The recovered magnetite nanoparticles were washed several times with acetone and then dried at room temperature (Fig. 8). The results of five consecutive runs (98%, 98%, 97%, 96% and 96%) indicated that the yields remained similar with no detectable loss of yield or catalytic activity.

**Fig. 9:**
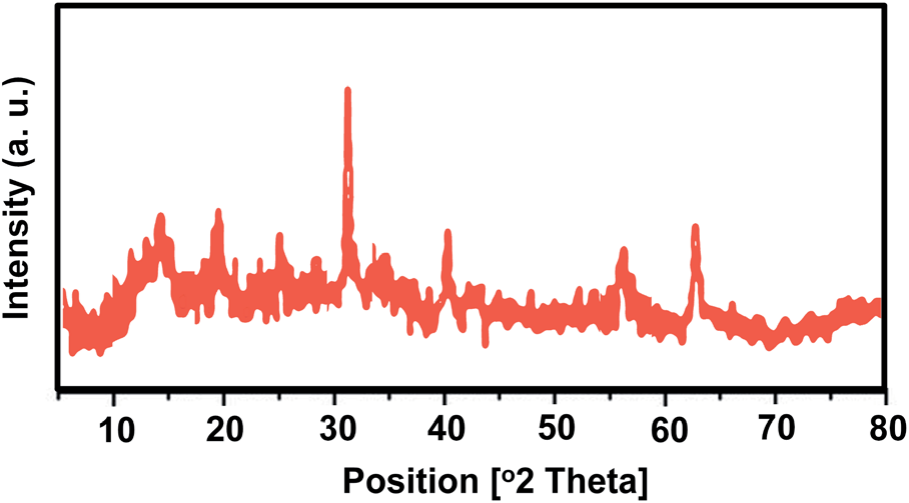
XRD pattern of recovered catalyst.

**Fig. 10:**
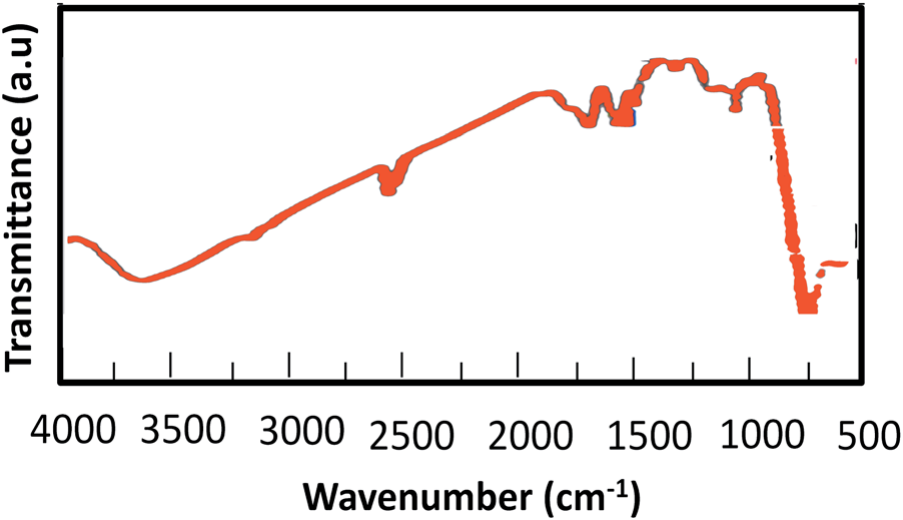
FT-IR spectrum of recovered catalyst.

In order to investigate any structural change of the catalyst, the XRD pattern (Fig. 9) and FT-IR spectrum (Fig. 10) of the recovered catalyst were obtained. The results show well-defined peaks at the 2*θ* values of the XRD pattern related to the unused catalyst and conformity of the FT-IR spectrum to that of the unused catalyst; thus, there is no structural change in the catalyst after the reaction.

## 3. Conclusions

In this research, we have developed an effective procedure for the synthesis of 5-phenyl-spiro[diindenopyridine-indenoquinoxaline]-dione derivatives by a one-pot four-component reaction of ninhydrin, 1,2-diamino benzene, 1,3-indandione and anilines using MnFe_2_O_4_@CS-Bu-SO_3_H MNPs as a nano magnetic catalyst. High yields, a simple reaction process, easy work-up, shorter reaction times, reusability of the catalyst and low catalyst loading are some of the considerable advantages of the current protocol.

## 4. Experimental method

### 4.1. Materials

The chemicals used in this work were obtained from Fluka and Merck Chemical Companies and were used without purification.

### 4.2. Apparatus

FT-IR spectra were obtained with potassium bromide pellets in the range 400–4000 cm^−1^ with a PerkinElmer 550 spectrometer. ^1^H NMR and ^13^C NMR spectra were recorded with a Bruker DRX-400 spectrometer at 400 and 100 MHz, respectively. NMR spectra were reported as parts per million (ppm) downfield from tetramethylsilane as the internal standard. The abbreviations used are singlet (s), doublet (d), triplet (t) and multiplet (m). Nanostructures were characterized using a Holland Philips Xpert X-ray powder diffraction (XRD) diffractometer (CuKα, radiation, *k* = 0.154056 nm) at a scanning speed of 2° min^−1^ from 10° to 100° (2*θ*). A BANDELIN ultrasonic HD 3200 with probe model KE 76 with a diameter of 6 mm was used to produce ultrasonic irradiation and homogenize the reaction mixture. The piezoelectric crystal of this kind of probe normally works in the range of 700 kHz, but by using the proper clamps, the output frequency of the piezoelectric crystal was controlled and reduced to 20 kHz. Therefore, the induced frequency of the probe for the reaction mixture is equal to 20 kHz. A thermal method was used for the calibration of ultrasonic power. Melting points were determined in open capillaries using an Electrothermal Mk3 apparatus and are uncorrected. The purity determination of the substrates and reaction monitoring were accomplished by TLC on silica-gel polygram SILG/UV 254 plates (Merck). FE-SEM images of the products were visualized by an Oxford Instruments Sigma ZEISS field emission scanning electron microscope. The magnetic properties of the nanoparticles were measured with a vibrating sample magnetometer (VSM, PPMS-9T) at 300 K in Iran (University of Kashan).

### 4.3. Typical experimental procedure for the preparation of chitosan-coated magnetic nanoparticles MnFe_2_O_4_@CS NPs

In this research, the MnFe_2_O_4_ nanoparticles were prepared by following the reported standard protocol: co-precipitation of MnCl_2_ and FeCl_3_ in water in the presence of sodium hydroxide. Briefly, MnCl_2_·4H_2_O and FeCl_3_·6H_2_O were used in the molar ratio of Mn^2+^ : Fe^3+^ = 1 : 2 to prepare a 0.4 mol L^−1^ metal ion solution of 100 ml containing 0.2 mol L^−1^ Mn^2+^ and 0.4 mol L^−1^ Fe^3+^ which was then dropped slowly into 100 ml NaOH solution of 5 mol L^−1^ at the temperature of 97 °C. After aging for 2 h with continuous stirring, the mixture was filtered, washed and dried at 60 °C for 24 h. Next, the reaction mixture was cooled and the catalyst was isolated in a magnetic field and washed three times with distilled water. Subsequently, in order to prepare MnFe_2_O_4_–chitosan nanoparticles, 1 g of the MnFe_2_O_4_ nanoparticles was dispersed in 120 ml distilled water under ultrasound irradiation and 0.5 g of chitosan in 120 ml of 2.0 wt% acetic acid solution was slowly added under vigorous stirring at 50 °C for 1 h. The modified MnFe_2_O_4_–chitosan nanoparticles were recovered by magnetic decantation and washed with CH_2_Cl_2_. Finally, MnFe_2_O_4_–chitosan nanoparticles were dried at 60 °C.

### 4.4. Preparation of 1,4-butane sultone chitosan-encapsulated manganese ferrite nanoparticles (MnFe_2_O_4_@CS-*n*-Bu-SO_3_H MNPs)

**Scheme 3:**
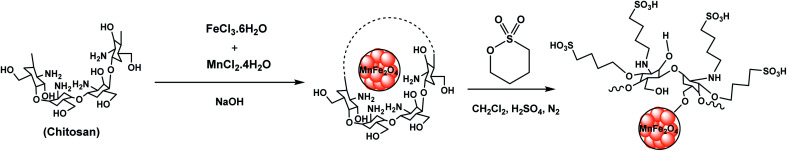
Preparation steps of MnFe_2_O_4_@CS-Bu-SO_3_H MNPs.

MnFe_2_O_4_–chitosan nanoparticles and 1,4-butane sultone (0.36 ml, 3 mmol) were mixed together without solvent and stirred for 3 h at room temperature (25 °C). Concentrated H_2_SO_4_ (0.154 ml, 3 mmol) was added to the above catalyst mixture and refluxed for 2 h. The final catalyst was washed repeatedly with ethanol and then dried (Scheme 3).

### 4.5. General procedure for the synthesis of spiro indenoquinoxalines catalyzed by MnFe_2_O_4_@CS-Bu-SO_3_H MNPs

A mixture of ninhydrin (1 mmol), 1,2-diamino benzene (1 mmol), and MnFe_2_O_4_@CS-Bu-SO_3_H MNPs (20 mg) in 5 ml acetonitrile was heated at 75 °C for 5 min, then 1,3-indandione (2 mmol) and aniline (1 mmol) were added to the mixture and stirred for 3 h. Progress of the reaction was continuously monitored by TLC. On completion of the reaction, the reaction mixture was cooled to room temperature and the catalyst was separated by an external magnet. Then, the precipitated product was filtered and washed in 3 ml ethanol to afford the pure product.

### 4.6. Representative spectral data

#### 4.6.1 5-(4-Methoxyphenyl)-10*H*-spiro[diindeno[1,2-*b*:2′,1′-*e*]pyridine-11,11′-indeno[1,2-*b*]quinoxaline]-10,12(5*H*)-dione (5a)

Dark red solid, Rf (*n*-hexane : ethylacetate): 7 : 3 (v/v) = 0.60; (mp_rep_. 233)/(mp_lit_ 233–240 °C).^6^ IR (KBr) *ν*: 3436, 3060, 2924, 1699, 1511, 1383, 1252, 1000 cm^−1^; ^1^H NMR (400 MHz, DMSO-d_6_) *δ*: 8.23 (d, *J* = 8.0 Hz, 1H, ArH), 8.18 (m, 2H, ArH), 7.99 (t, *J* = 8.0 Hz, 2H, ArH), 7.87 (d, *J* = 8.0 Hz, 1H, ArH), 7.78 (d, *J* = 8.0 Hz, 2H, ArH), 7.60 (t, *J* = 4.0 Hz, 2H, ArH), 7.33 (m, 2H, ArH), 7.22 (m, 4H, ArH), 7.09 (d, *J* = 8.0 Hz, 2H, ArH), 5.97 (d, *J* = 4.0 Hz, 2H, ArH), 3.98 (s, 3H, OCH_3_) ppm. Anal. calcd for C_40_H_23_N_3_O_3_: C, 80.93; H, 3.91; N, 7.08. Found: C, 80.88; H, 3.86; N, 7.04.

#### 4.6.2 5-(3-Chlorophenyl)-10*H*-spiro[diindeno[1,2-*b*:2′,1′-*e*]pyridine-11,11′-indeno[1,2-*b*]quinoxaline]-10,12(5*H*)-dione (5b)

Dark red solid, Rf (*n*-hexane : ethylacetate): 7 : 3 (v/v) = 0.55; mp 284 °C. IR (KBr) *ν*: 3432, 3063, 2922, 1700, 1578, 1384, 1301, 999 cm^−1^; ^1^H NMR (400 MHz, DMSO-d_6_) *δ*: 8.22 (d, *J* = 8.0 Hz, 1H, ArH), 8.17 (d, *J* = 4.0 Hz, 2H, ArH), 8.13 (t, *J* = 8.0 Hz, 1H, ArH), 8.02 (d, *J* = 8.0 Hz, 2H, ArH), 7.86 (m, 3H, ArH), 7.77 (s, 1H, ArH), 7.60 (d, *J* = 4.0 Hz, 2H, ArH), 7.23 (m, 4H, ArH), 7.11 (d, *J* = 4.0 Hz, 2H, ArH), 5.60 (d, *J* = 4.0 Hz, 2H, ArH) ppm; ^13^C NMR (100 MHz, DMSO-d_6_): *δ* 46.2, 113.4, 121.7, 122.1, 122.8, 124.7, 129.4, 129.6, 129.7, 129.8, 130.3, 130.9, 131.4, 132.4, 132.7, 132.9, 133.2, 136.5, 136.6, 137.4, 139.5, 141.5, 142.3, 151.9, 155.0, 156.6, 156.7, 190.1 ppm. Anal. calcd for C_39_H_20_ClN_3_O_2_: C, 78.33; H, 3.37; N, 7.03. Found: C, 78.28; H, 3.33; N, 6.96.

#### 4.6.3 5-(4-Hydroxyphenyl)-10*H*-spiro[diindeno[1,2-*b*:2′,1′-*e*]pyridine-11,11′-indeno[1,2-*b*]quinoxaline]-10,12(5*H*)-dione (5c)

Dark red solid, Rf (*n*-hexane : ethylacetate): 7 : 3 (v/v) = 0.51; mp: 251 °C. IR (KBr) *ν*: 3431, 3041, 2938, 1728, 1568, 1382, 1335, 1198 cm^−1^; ^1^H NMR (400 MHz, DMSO-d_6_) *δ*: 8.18 (s, 1H, ArH), 8.15 (s, 1H, ArH), 8.14 (s, 1H, ArH), 8.12 (s, *J* = 8.0 Hz, 1H, ArH), 8.07 (s, 1H, ArH), 7.91 (s, 1H, ArH), 7.89 (s, 1H, ArH), 7.86 (s, 3H, ArH), 7.72 (s, 1H, ArH), 7.70 (s, 1H, ArH), 7.68 (s, 1H, ArH), 7.30 (s, 1H, ArH), 7.29 (s, 1H, ArH), 7.25 (s, 1H, ArH), 7.21 (s, 1H, ArH), 7.08 (s, 1H, OH), 5.71 (d, *J* = 4.0 Hz, 2H, ArH) ppm. Anal. calcd for C_39_H_21_N_3_O_3_: C, 80.82; H, 3.65; N, 7.25. Found: C, 80.75; H, 3.60; N, 7.19.

#### 4.6.4 5-(4-Chlorophenyl)-10*H*-spiro[diindeno[1,2-*b*:2′,1′-*e*]pyridine-11,11′-indeno[1,2-*b*]quinoxaline]-10,12(5*H*)-dione (5d)

Dark red solid, Rf (*n*-hexane : ethylacetate): 7 : 3 (v/v) = 0.63; mp 289 °C. IR (KBr) *ν*: 3431, 3042, 2923, 1728, 1569, 1383, 1337, 1197 cm^−1^; ^1^H NMR (400 MHz, DMSO-d_6_) *δ*:^1^H NMR (400 MHz, DMSO-d_6_): *δ* (ppm) 8.18 (s, 1H, ArH), 8.15 (s, 1H, ArH), 8.14 (s, 1H, ArH), 8.12 (s, 1H, ArH), 8.09 (s, 2H, ArH), 7.95 (s, 1H, ArH), 7.89 (s, 3H, ArH), 7.87 (s, 2H, ArH), 7.70 (t, *J* = 8.0 Hz, 2H, ArH), 7.22 (s, 1H, ArH), 7.20 (s, 1H, ArH), 7.10 (d, *J* = 4.0 Hz, 3H, ArH), 5.65 (d, *J* = 4.0 Hz, 2H, ArH) ppm. Anal. calcd for C_39_H_20_ClN_3_O_2_: C, 78.33; H, 3.37; N, 7.03. Found: C, 78.26; H, 3.30; N, 6.98.

#### 4.6.5 5-(3-Nitrophenyl)-10*H*-spiro[diindeno[1,2-*b*:2′,1′-*e*]pyridine-11,11′-indeno[1,2-*b*]quinoxaline]-10,12(5*H*)-dione (5e)

Dark red solid, Rf (*n*-hexane : ethylacetate): 7 : 3 (v/v) = 0.58; mp 181 °C. IR (KBr) *ν*: 3439, 3067, 2911, 1701, 1535, 1383, 1343, 1000 cm^−1^; ^1^H NMR (400 MHz, DMSO-d_6_) *δ*: 8.24 (d, *J* = 8.0 Hz, 2H, ArH), 8.18 (d, *J* = 8.0 Hz, 1H, ArH), 8.11 (m, 3H, ArH), 7.88 (m, 3H, ArH), 7.63 (t, *J* = 8.0 Hz, 3H, ArH), 7.24 (d, *J* = 8.0 Hz, 2H, ArH), 7.14 (m, 4H, ArH), 5.97 (d, *J* = 8.0 Hz, 2H, ArH) ppm. Anal. calcd for C_39_H_20_N_4_O_5_: C, 75.00; H, 3.23; N, 8.97. Found: C, 74.95; H, 3.18; N, 8.91.

#### 4.6.6 5-(2-Chlorophenyl)-10*H*-spiro[diindeno[1,2-*b*:2′,1′-*e*]pyridine-11,11′-indeno[1,2-*b*]quinoxaline]-10,12(5*H*)-dione (5f)

Dark red solid, Rf (*n*-hexane : ethylacetate): 7 : 3 (v/v) = 0.62; mp 281 °C. IR (KBr) *ν*: 3435, 3061, 2922, 1703, 1578, 1386, 1337, 1003 cm^−1^; ^1^H NMR (400 MHz, DMSO-d_6_) *δ*: 8.19 (m, 1H, ArH), 8.15 (m, 1H, ArH), 8.06 (m, 1H, ArH), 8.02 (m, 1H, ArH), 7.94 (m, 1H, ArH), 7.87 (m, 3H, ArH), 7.78 (m, 2H, ArH), 7.73 (m, 1H, ArH), 7.62 (m, 1H, ArH), 7.54 (m, 1H, ArH), 7.27 (m, 1H, ArH), 7.18 (m, 1H, ArH), 7.12 (m, 1H, ArH), 7.05 (m, 1H, ArH), 6.94 (m, 1H, ArH), 5.59 (d, *J* = 4.0 Hz, 2H, ArH) ppm. Anal. calcd for C_39_H_20_ClN_3_O_2_: C, 78.33; H, 3.37; N, 7.03. Found: C, 78.27; H, 3.29; N, 6.98.

#### 4.6.7 5-Phenyl-10*H*-spiro[diindeno[1,2-*b*:2′,1′-*e*]pyridine-11,11′-indeno[1,2-*b*]quinoxaline]-10,12(5*H*)-dione (5g)

Dark red solid, Rf (*n*-hexane : ethylacetate): 7 : 3 (v/v) = 0.64; (mp_rep_. 277 °C)/(mp_lit_ > 280 °C).^6^ IR (KBr) *ν*: 3430, 3062, 2910, 1696, 1579, 1385, 1336, 999 cm^−1^; ^1^H NMR (400 MHz, DMSO-d_6_) *δ*: 8.88 (m, 3H, ArH), 8.05 (m, 3H, ArH), 7.92 (m, 2H, ArH), 7.83 (m, 3H, ArH), 7.62 (m, 2H, ArH), 7.23 (m, 2H, ArH), 7.10 (m, 4H, ArH), 5.59 (d, *J* = 8.0 Hz, 2H, ArH) ppm. Anal. calcd for C_39_H_21_N_3_O_2_: C, 83.11; H, 3.76; N, 7.46. Found: C, 83.07; H, 3.69; N, 7.38.

#### 4.6.8 5-(*o*-Tolyl)-10*H*-spiro[diindeno[1,2-*b*:2′,1′-*e*]pyridine-11,11′-indeno[1,2-*b*]quinoxaline]-10,12(5*H*)-dione (5h)

Dark red solid, Rf (*n*-hexane : ethylacetate): 7 : 3 (v/v) = 0.61; mp 201 °C. IR (KBr) *ν*: 3438, 3061, 2927, 1697, 1578, 1386, 1337, 1000 cm^−1^; ^1^H NMR (400 MHz, DMSO-d_6_) *δ*: 8.21 (m, 2H, ArH), 8.19 (m, 1H, ArH), 8.02 (m, 2H, ArH), 7.86 (m, 2H, ArH), 7.83 (m, 2H, ArH), 7.77 (m, 2H, ArH), 7.67 (m, 1H, ArH), 7.23 (m, 1H, ArH), 7.21 (m, 1H, ArH), 7.15 (m, 1H, ArH), 7.12 (m, 1H, ArH), 6.96 (m, 1H, ArH), 6.88 (m, 1H, ArH), 5.55 (d, *J* = 8.0 Hz, 2H, ArH), 1.21 (s, 3H, CH_3_) ppm; ^13^C NMR (100 MHz, DMSO-d_6_) *δ*: 17.7, 46.4, 121.9, 122.1, 122.8, 124.7, 128.6, 129.1, 129.5, 129.8, 130.3, 130.8, 130.0, 131.4, 132.1, 132.4, 132.9, 133.2, 133.3, 136.4, 136.8, 137.1, 137.4, 138.2, 141.5, 142.6, 151.9, 154.9, 156.8, 156.2, 190.1 ppm. Anal. calcd for C_40_H_23_N_3_O_2_: C, 83.17; H, 4.01; N, 7.27. Found: C, 83.11; H, 3.96; N, 7.17.

#### 4.6.9 5-(2,4-Dimethylphenyl)-10*H*-spiro[diindeno[1,2-*b*:2′,1′-*e*]pyridine-11,11′-indeno[1,2-*b*]quinoxaline]-10,12(5*H*)-dione (5i)

Dark red solid, Rf (*n*-hexane : ethylacetate): 7 : 3 (v/v) = 0.58; mp 234 °C. IR (KBr) *ν*: 3428, 3062, 2923, 1702, 1573, 1385, 1337, 999 cm^−1^; ^1^H NMR (400 MHz, DMSO-d_6_) *δ*: 8.18 (d, *J* = 8.0 Hz, 1H, ArH), 8.15 (s, 1H, ArH), 8.13 (s, 2H, ArH), 8.01 (d, *J* = 8.0 Hz, 1H, ArH), 7.92 (s, 2H, ArH), 7.89 (d, *J* = 8.0 Hz, 2H, ArH), 7.73 (d, *J* = 8.0 Hz, 2H, ArH), 7.61 (s, 1H, ArH), 7.52 (s, 1H, ArH), 7.46 (d, *J* = 8.0 Hz, 1H, ArH), 7.22 (d, *J* = 8.0 Hz, 2H, ArH), 7.16 (d, *J* = 8.0 Hz, 1H, ArH), 7.08 (s, 2H, ArH), 5.63 (d, *J* = 4.0 Hz, 2H, ArH), 2.56 (s, 3H, CH_3_), 2.38 (s, 3H, CH_3_) ppm. Anal. calcd for C_41_H_25_N_3_O_2_: C, 83.23; H, 4.26; N, 7.10. Found: C, 83.18; H, 4.19; N, 7.01.

#### 4.6.10 5-(4-Methylphenyl)-10*H*-spiro[diindeno[1,2-*b*:2′,1′-*e*]pyridine-11,11′-indeno[1,2-*b*]quinoxaline]-10,12(5*H*)-dione (5j)

Dark red solid, Rf (*n*-hexane : ethylacetate): 7 : 3 (v/v) = 0.64; (mp_rep_. 197 °C)/(mp_lit_ 193–205 °C).^6^ IR (KBr) *ν*: 3432, 3061, 2923, 1697, 1577, 1386, 1337, 997 cm^−1^; ^1^H NMR (400 MHz, DMSO-d_6_) *δ*: 8.24 (s, 1H, ArH), 8.19 (d, *J* = 8.0 Hz, 1H, ArH), 8.13 (s, 1H, ArH), 8.05 (d, *J* = 8.0 Hz, 3H, ArH), 7.92 (d, *J* = 8.0 Hz, 1H, ArH), 7.87 (t, *J* = 4.0 Hz, 2H, ArH), 7.79 (d, *J* = 8.0 Hz, 1H, ArH), 7.73 (d, *J* = 8.0 Hz, 2H, ArH), 7.60 (d, *J* = 4.0 Hz, 2H, ArH), 7.24 (s, 1H, ArH), 7.22 (d, *J* = 8.0 Hz, 1H, ArH), 7.16 (t, *J* = 8.0 Hz, 1H, ArH), 7.09 (d, *J* = 4.0 Hz, 1H, ArH), 5.97 (d, *J* = 8.0 Hz, 2H, ArH), 1.22 (s, 3H, CH_3_) ppm; Anal. calcd for C_40_H_23_N_3_O_2_: C, 83.17; H, 4.01; N, 7.27. Found: C, 83.09; H, 3.93; N, 7.18.

#### 4.6.11 5-(4-Nitrophenyl)-10*H*-spiro[diindeno[1,2-*b*:2′,1′-*e*]pyridine-11,11′-indeno[1,2-*b*]quinoxaline]-10,12(5*H*)-dione (5k)

Dark red solid, Rf (*n*-hexane : ethylacetate): 7 : 3 (v/v) = 0.56; (mp_rep_. 178 °C)/(mp_lit_ 175–180 °C).^6^ IR (KBr) *ν*: 3431, 3065, 2923, 1705, 1578, 1339, 1308, 1001 cm^−1^; ^1^H NMR (400 MHz, DMSO-d_6_) *δ*: 8.24 (m, 1H, ArH), 8.22 (m, 1H, ArH), 8.18 (m, 1H, ArH), 8.09 (m, 2H, ArH), 7.93 (m, 2H, ArH), 7.89 (m, 1H, ArH), 7.77 (m, 1H, ArH), 7.75 (m, 1H, ArH), 7.32 (m, 2H, ArH), 7.23 (m, 1H, ArH), 7.11 (m, 1H, ArH), 5.63 (d, *J* = 8.0 Hz, 1H, ArH) ppm; Anal. calcd for C_39_H_20_N_4_O_4_: C, 76.97; H, 3.31; N, 9.21. Found: C, 76.90; H, 3.27; N, 9.15.

## Conflicts of interest

There are no conflicts to declare.

## Supplementary Material

RA-010-D0RA04925E-s001
