# Supplementary material for: Chitosan-encapsulated manganese ferrite particles bearing sulfonic acid group catalyzed efficient synthesis of spiro indenoquinoxalines
Source: RSC Adv. 2020 Sep 9;10(55):33334–43. doi: 10.1039/d0ra04925e (PMC9056677; doi:10.1039/d0ra04925e)
Supplement: RA-010-D0RA04925E-s001 [file RA-010-D0RA04925E-s001.pdf]

Electronic Supplementary Material (ESI) for RSC Advances.
This journal is © The Royal Society of Chemistry 2020

SUPPLEMENTARY INFORMATION

Manganese ferrite particles encapsulated-chitosan bearing sulfonic acid group catalyzed efficient synthesis of spiro indenoquinoxalines

Authors: Sepideh Lahouti and Hossein Naeimi*

NMR data

^{1}H NMR spectra of 5-(4-methoxyphenyl)-10H-spiro[diindeno[1,2-b:2',1'-e]pyridine-11,11'-indeno[1,2-b]quinoxaline]-10,12(5H)-dione (5a)

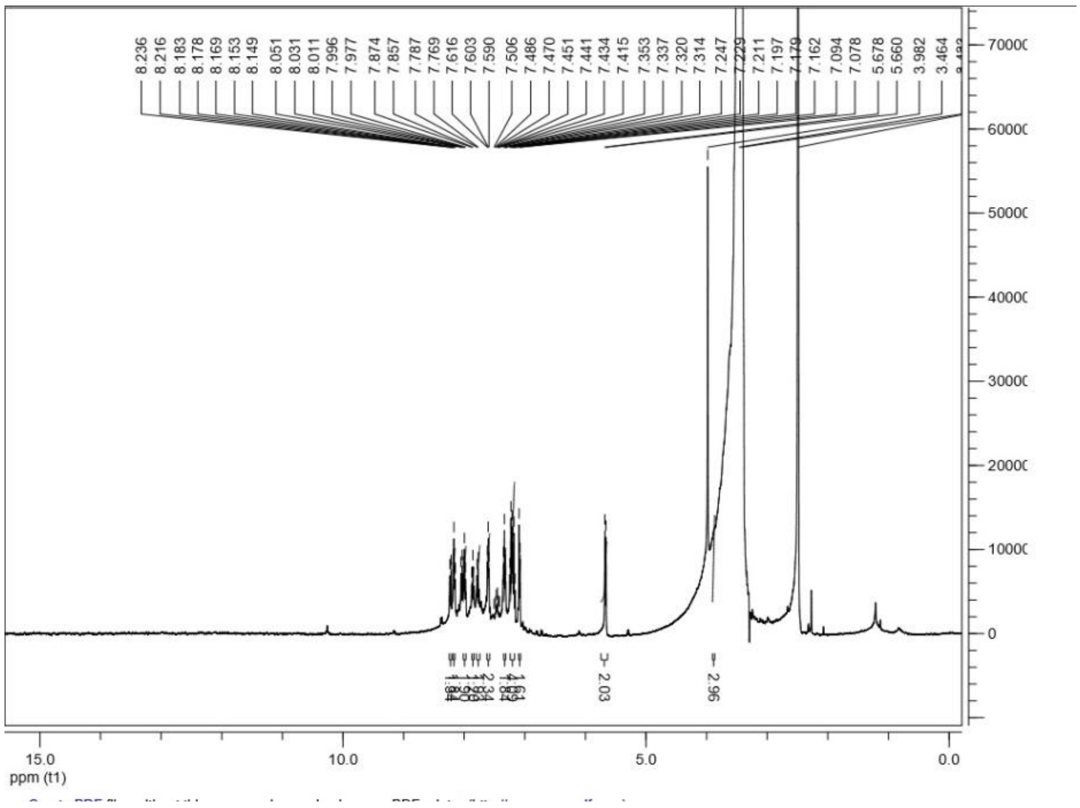

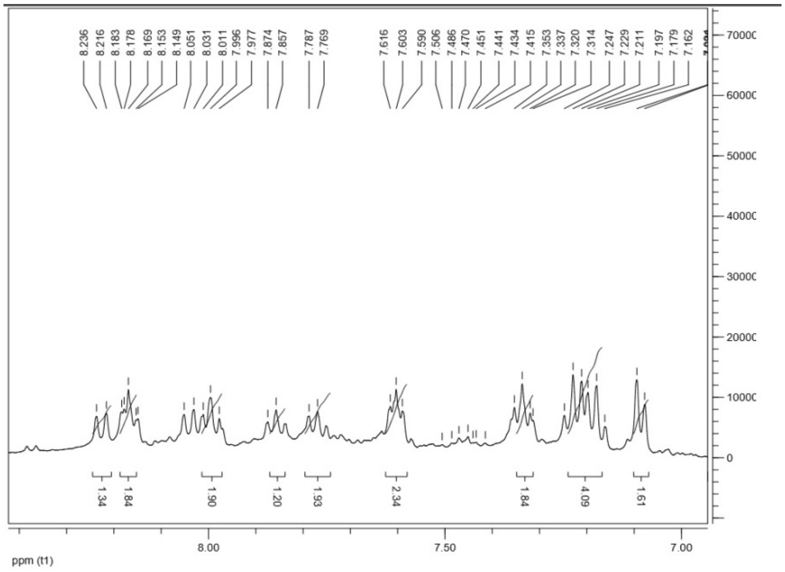

^{1}H NMR spectra of 5-(3-chlorophenyl)-10H-spiro[diindeno[1,2-b:2',1'-e]pyridine-11,11'-indeno[1,2-b]quinoxaline]-10,12(5H)-dione (5b)

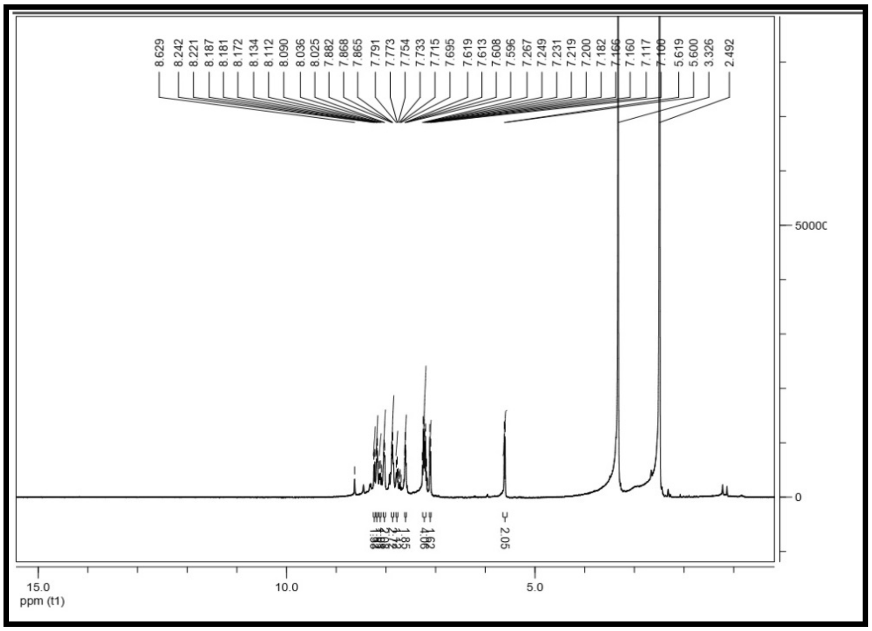

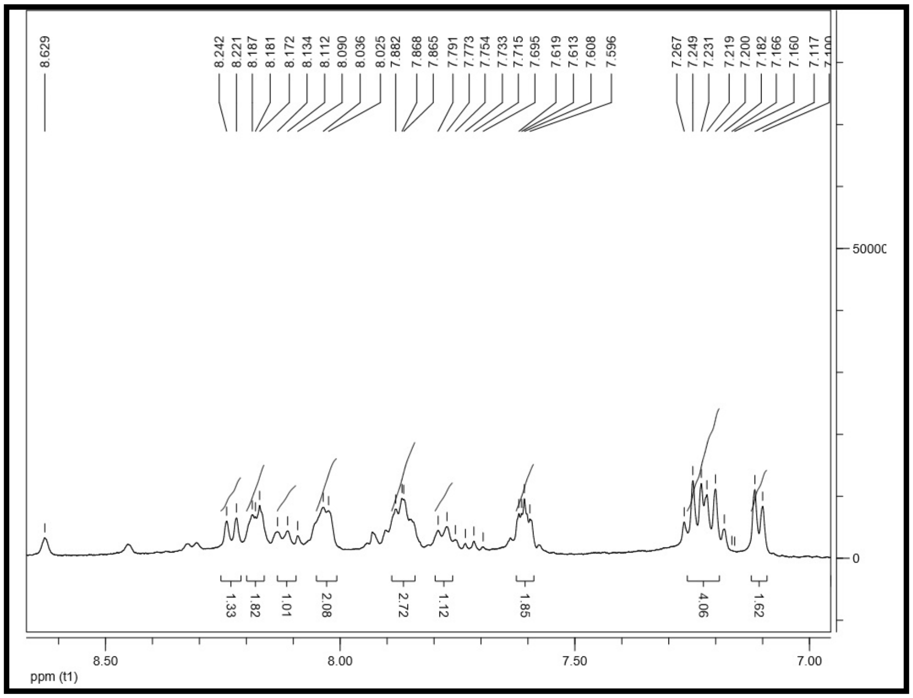

^{13}C NMR spectra of 5-(3-chlorophenyl)-10H-spiro[diindeno[1,2-b:2',1'-e]pyridine-11,11'-indeno[1,2-b]quinoxaline]-10,12(5H)-dione (5b)

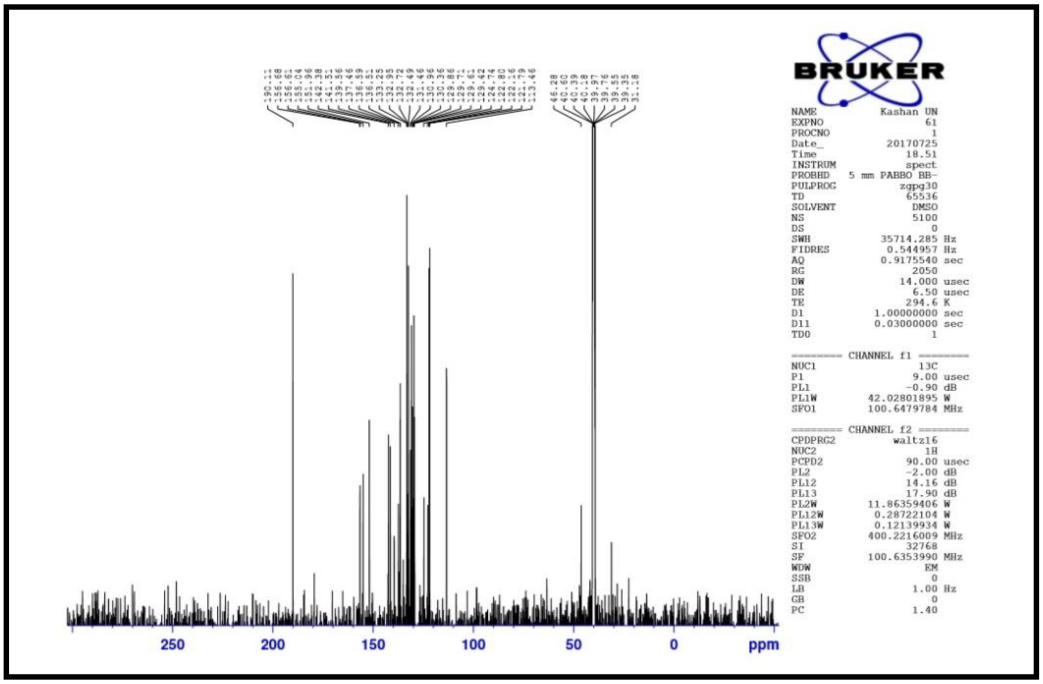

^{1}H NMR spectra of 5-(4-hydroxyphenyl)-10H-spiro[diindeno[1,2-b:2',1'-e]pyridine-11,11'-indeno[1,2-b]quinoxaline]-10,12(5H)-dione (5c)

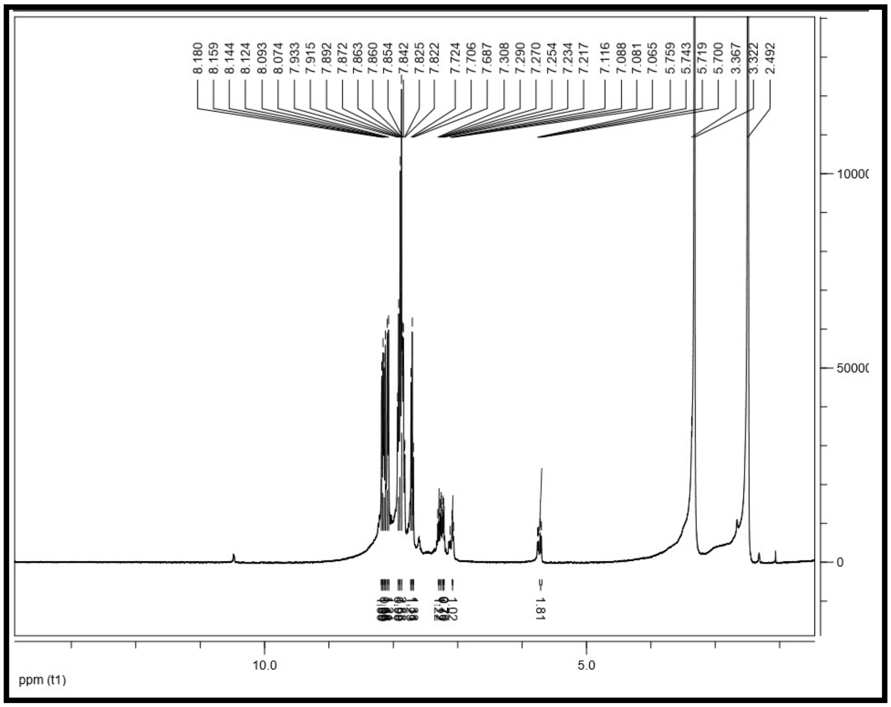

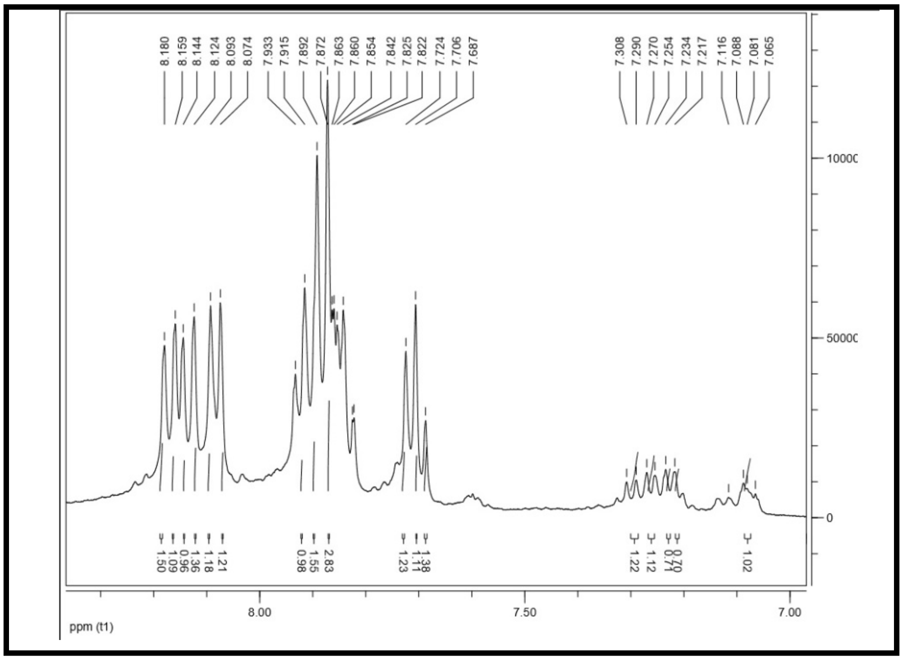

^{1}H NMR spectra of 5-(4-chlorophenyl)-10H-spiro[diindeno[1,2-b:2',1'-e]pyridine-11,11'-indeno[1,2-b]quinoxaline]-10,12(5H)-dione (5d)

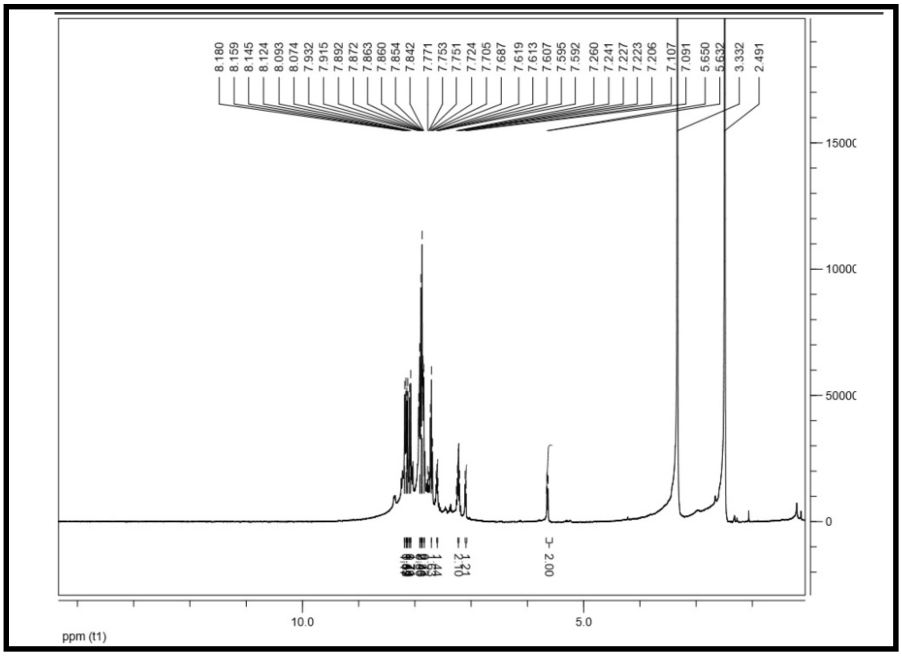

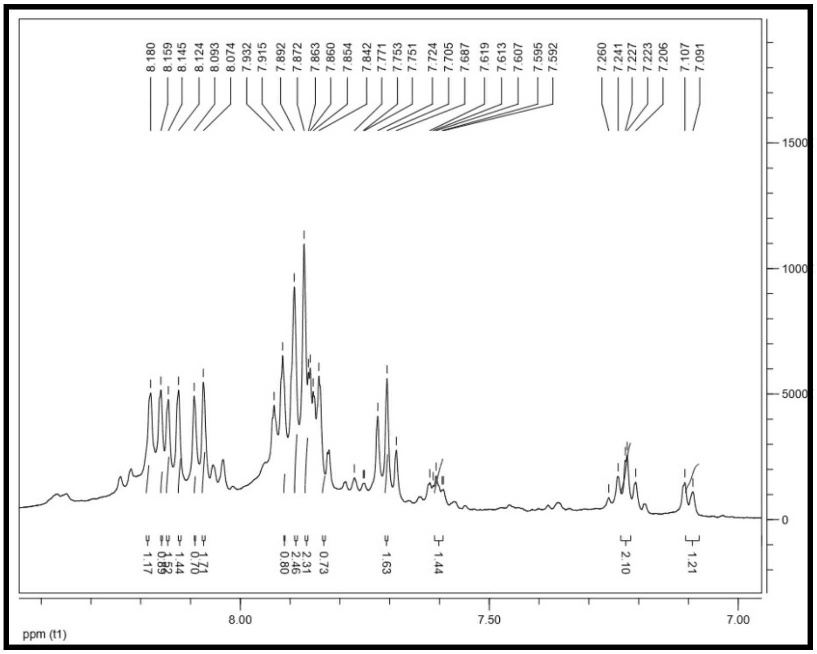

^{1}H NMR spectra of 5-(3-nitrophenyl)-10H-spiro[diindeno[1,2-b:2',1'-e]pyridine-11,11'-indeno[1,2-b]quinoxaline]-10,12(5H)-dione (5e)

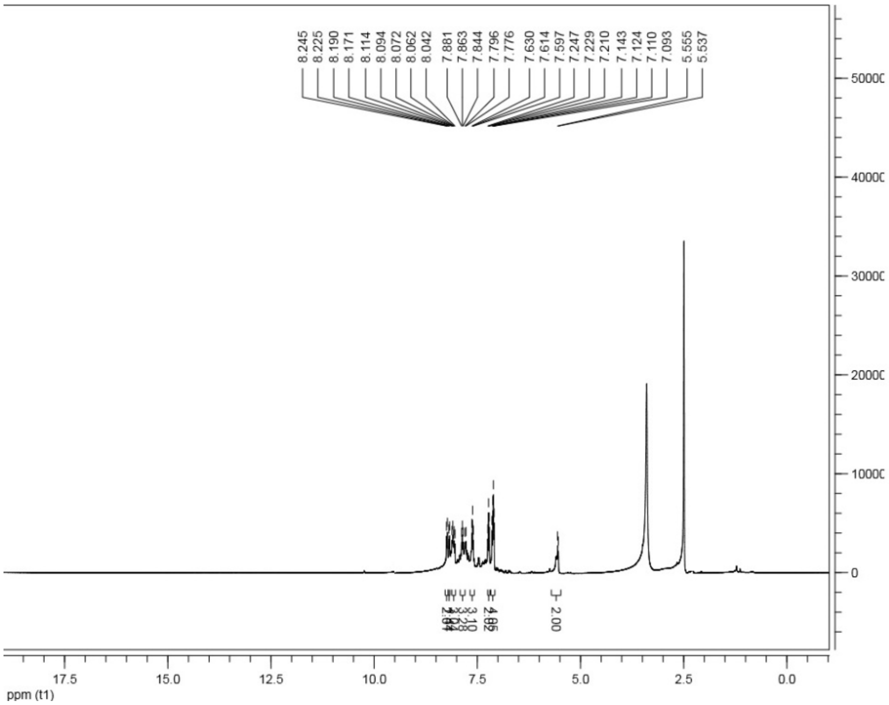

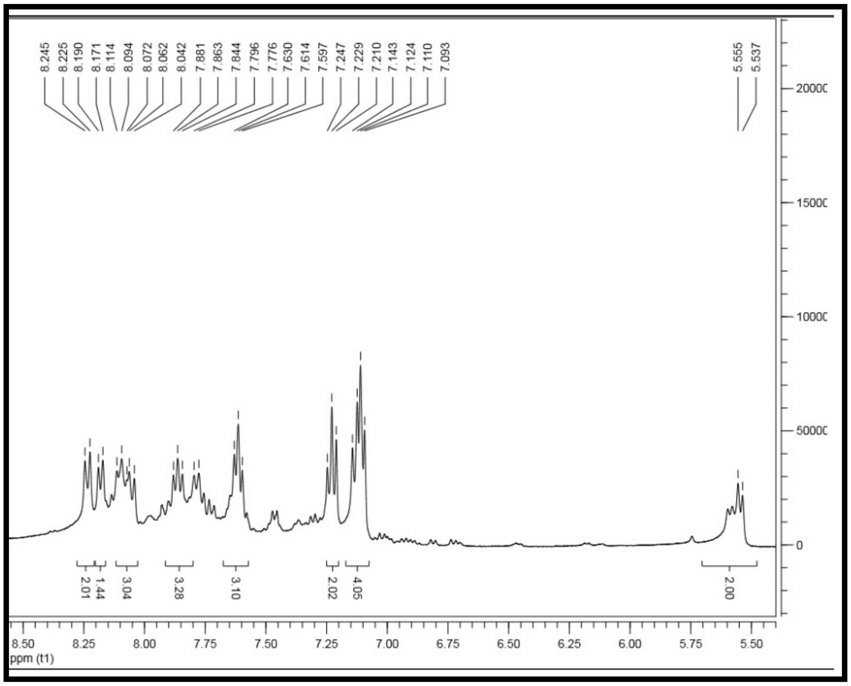

^{1}H NMR spectra of 5-(2-chlorophenyl)-10H-spiro[diindeno[1,2-b:2',1'-e]pyridine-11,11'-indeno[1,2-b]quinoxaline]-10,12(5H)-dione (5f)

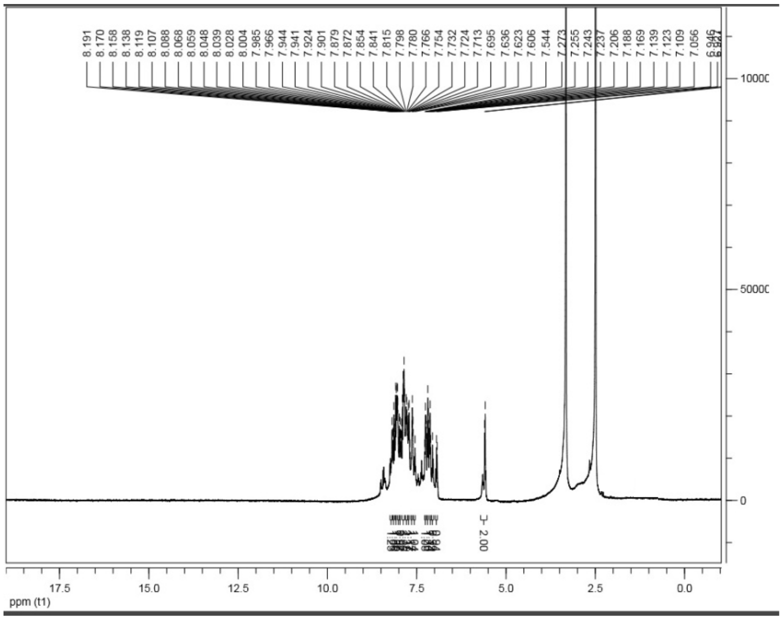

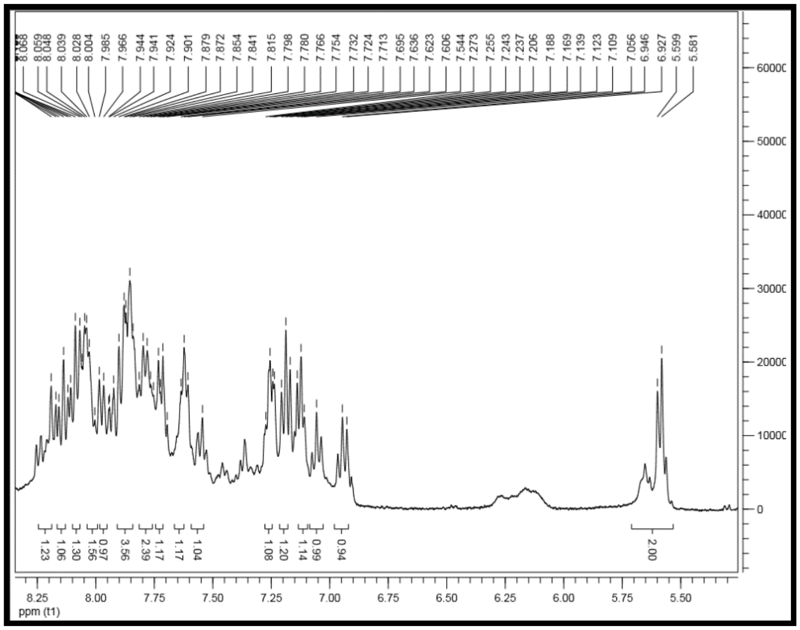

^{1}H NMR spectra of 5-phenyl-10H-spiro[diindeno[1,2-b:2',1'-e]pyridine-11,11'-indeno[1,2-b]quinoxaline]-10,12(5H)-dione (5g)

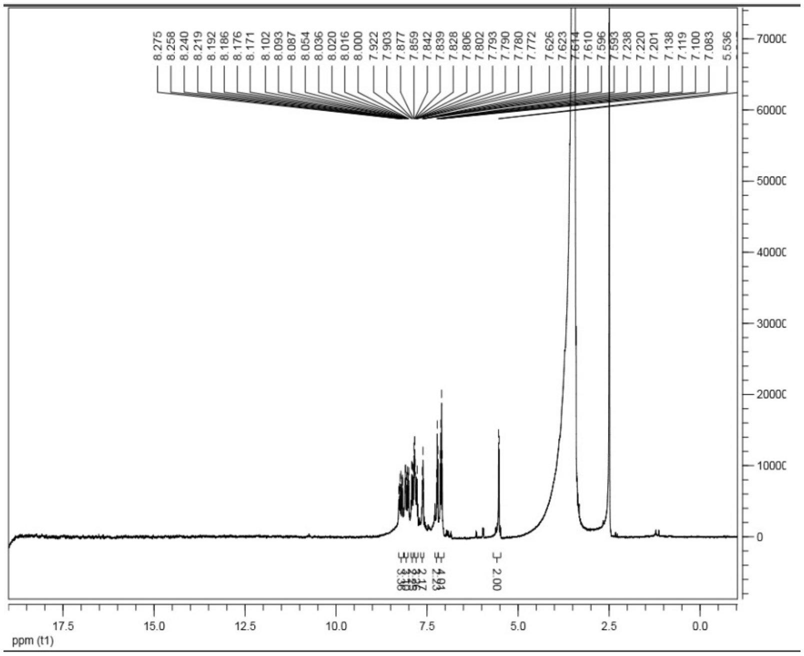

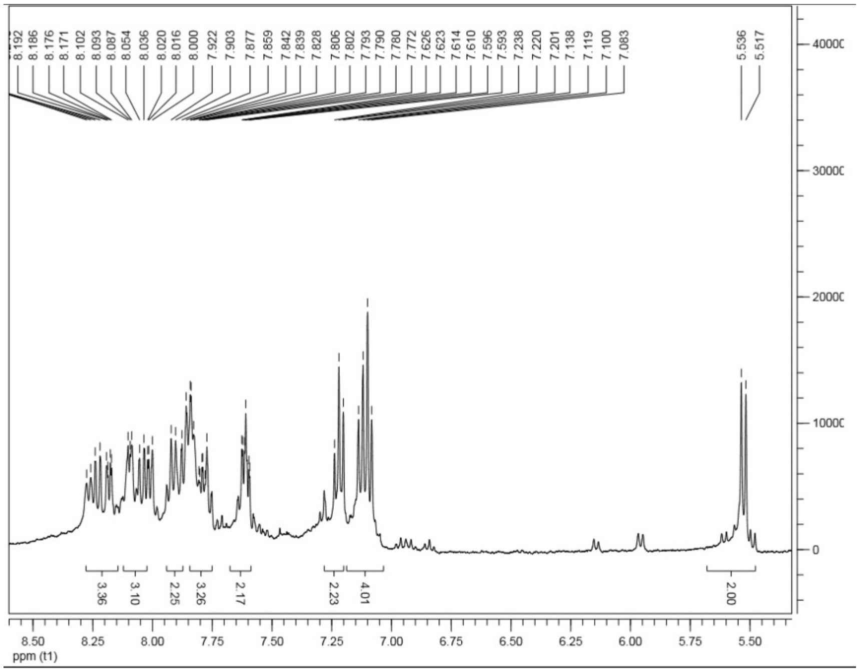

^{1}H NMR spectra of 5-(o-tolyl)-10H-spiro[diindeno[1,2-b:2',1'-e]pyridine-11,11'-indeno[1,2-b]quinoxaline]-10,12(5H)-dione (5h)

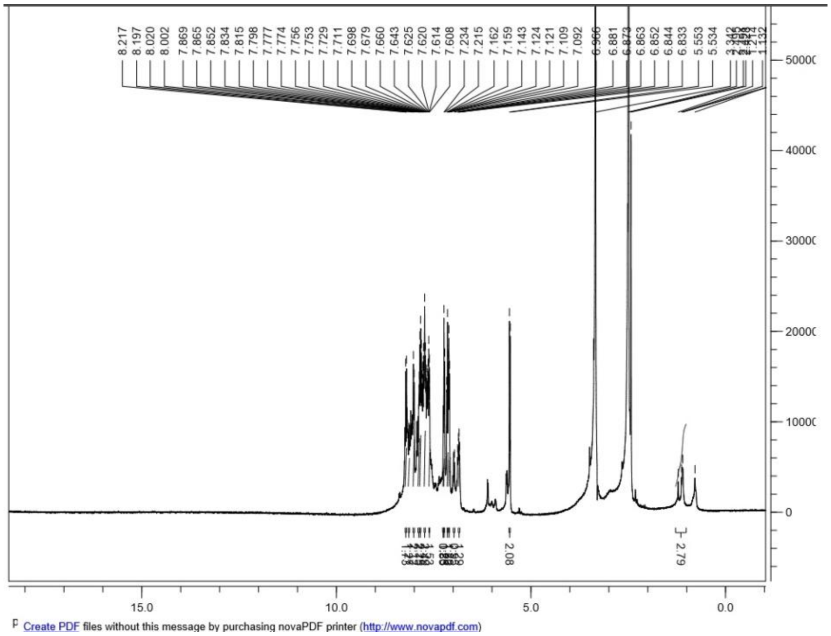

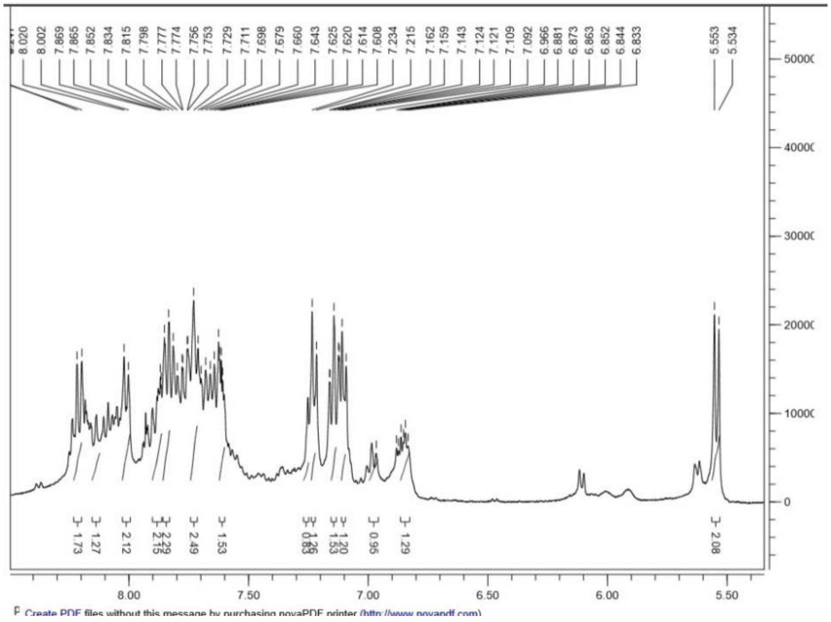

^{13}C NMR spectra of5-(o-tolyl)-10H-spiro[diindeno[1,2-b:2',1'-e]pyridine-11,11'-indeno[1,2-b]quinoxaline]-10,12(5H)-dione (5h)

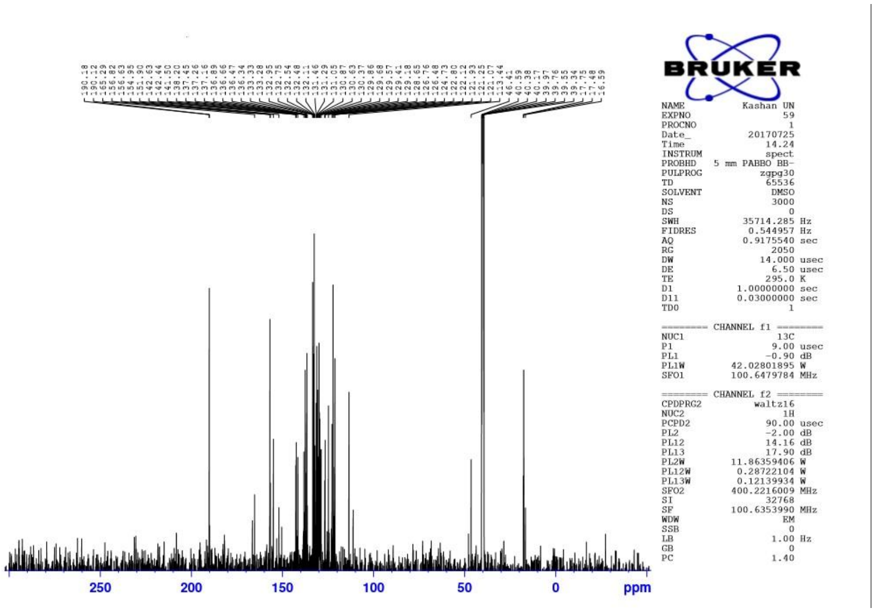

^{1}H NMR spectra of 5-(2,4-dimethylphenyl)-10H-spiro[diindeno[1,2-b:2',1'-e]pyridine-11,11'-indeno[1,2-b]quinoxaline]-10,12(5H)-dione (5i)

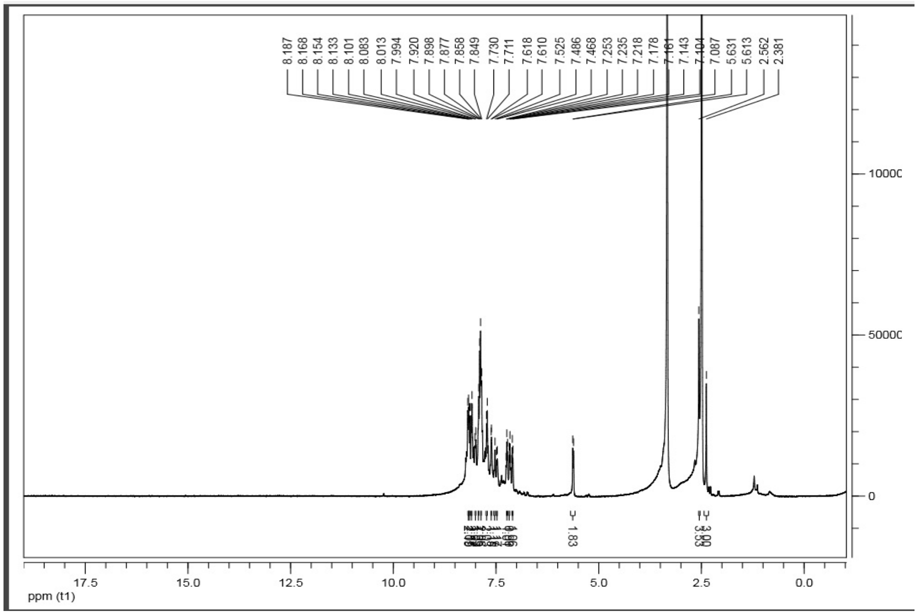

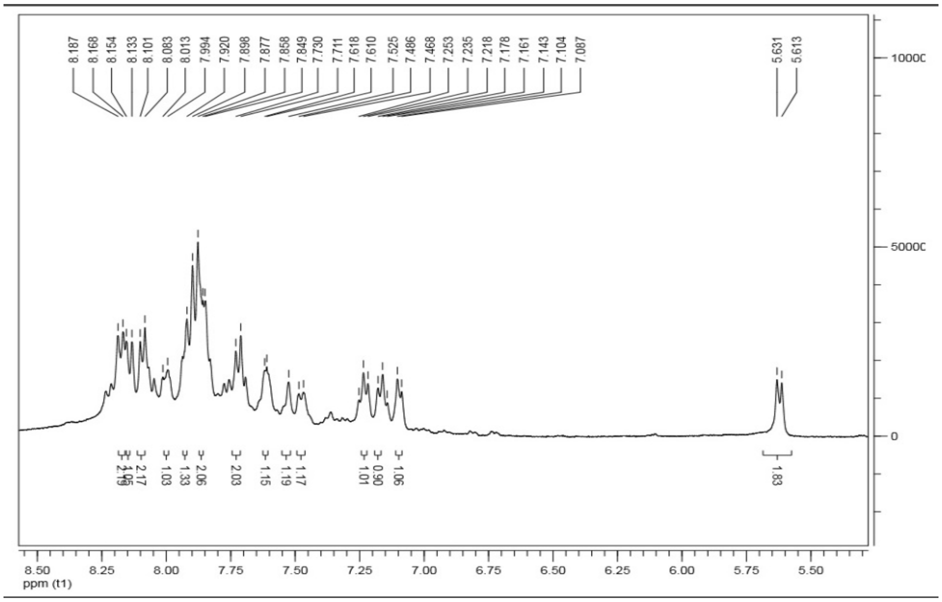

^{1}H NMR spectra of 5-(4-methylphenyl)-10H-spiro[diindeno[1,2-b:2',1'-e]pyridine-11,11'-indeno[1,2-b]quinoxaline]-10,12(5H)-dione (5k)

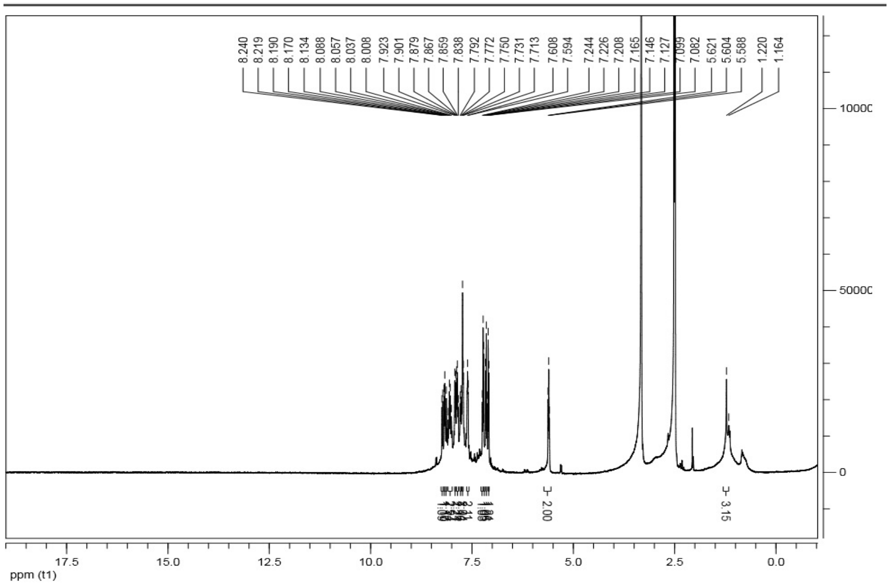

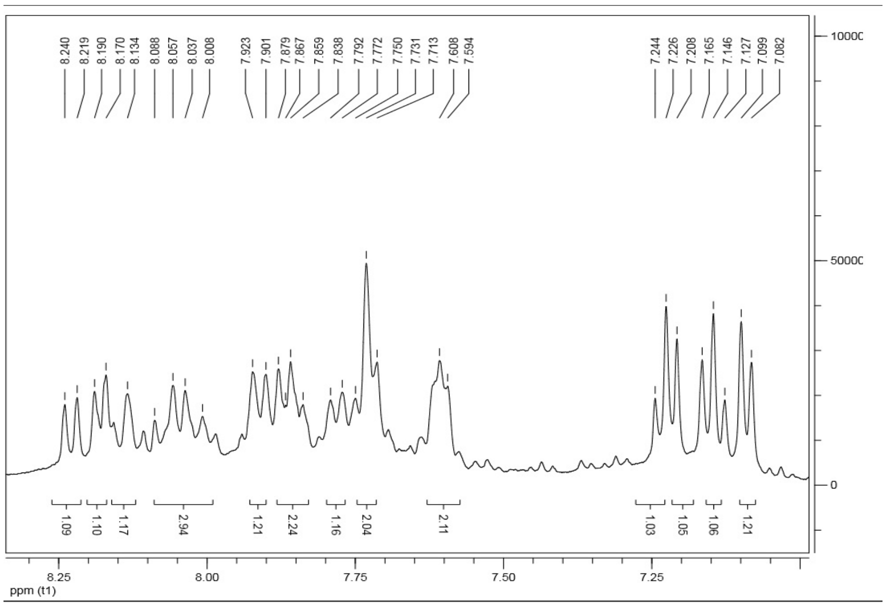

^{1}H NMR spectra of 5-(4-nitrophenyl)-10H-spiro[diindeno[1,2-b:2',1'-e]pyridine-11,11'-indeno[1,2-b]quinoxaline]-10,12(5H)-dione (5m)

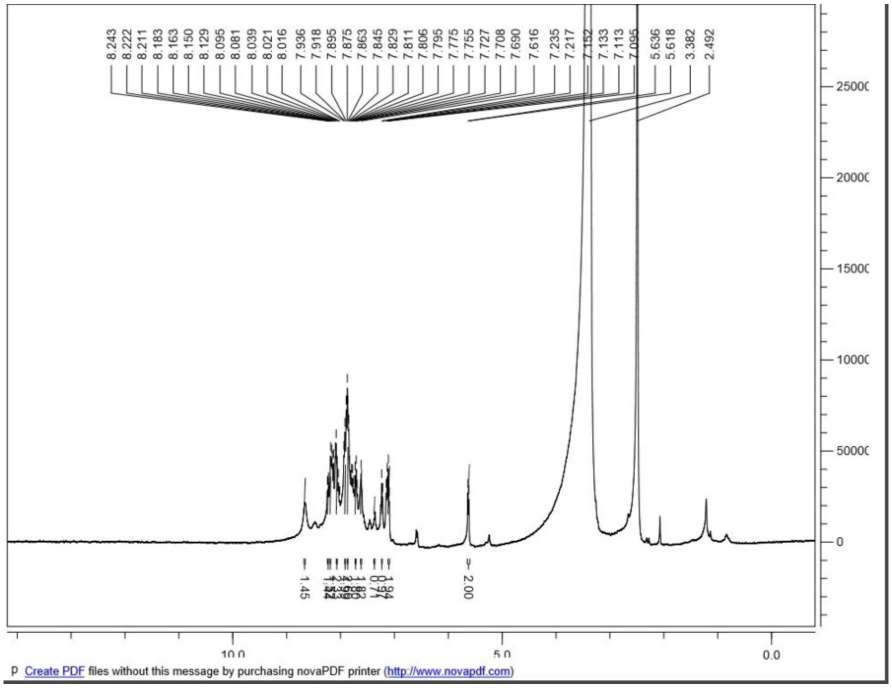
8.243
8.222
8.211
8.183
8.163
8.150
8.129
8.095
8.081
8.039
8.021
8.016
7.936
7.918
7.895
7.875
7.863
7.845
7.829
7.811
7.806
7.795
7.775
7.755
7.727
7.708
7.690
7.616
7.235
7.217
7.152
7.133
7.113
7.095
5.636
5.618
3.382
2.492
1.45
2.00
10.0
5.0
0.0
25000
20000
15000
10000
50000
0
p Create PDF files without this message by purchasing novaPDF printer (http://www.novapdf.com)

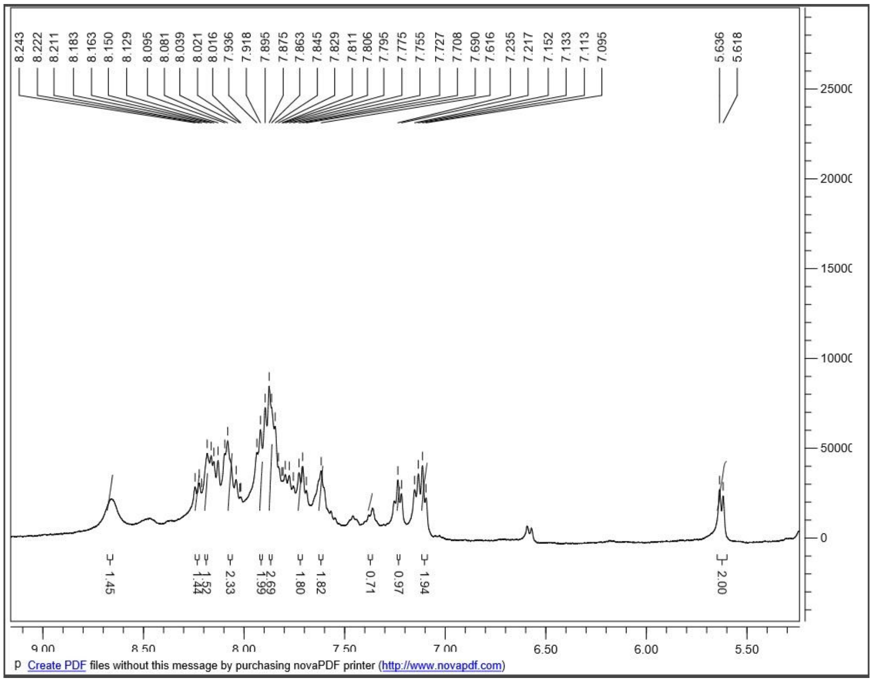
8.243
8.222
8.211
8.183
8.163
8.150
8.129
8.095
8.081
8.039
8.021
8.016
7.936
7.918
7.895
7.875
7.863
7.845
7.829
7.811
7.806
7.795
7.775
7.755
7.727
7.708
7.690
7.616
7.235
7.217
7.152
7.133
7.113
7.095
5.636
5.618
1.45
1.52
1.44
2.33
1.99
2.69
1.80
1.82
0.71
0.97
1.94
2.00
9.00
8.50
8.00
7.50
7.00
6.50
6.00
5.50
25000
20000
15000
10000
50000
0
p Create PDF files without this message by purchasing novaPDF printer (http://www.novapdf.com)
